# Smoking and the widening inequality in life expectancy between metropolitan and nonmetropolitan areas of the United States

**DOI:** 10.3389/fpubh.2022.942842

**Published:** 2022-09-07

**Authors:** Arun S. Hendi, Jessica Y. Ho

**Affiliations:** ^1^Office of Population Research and Department of Sociology, Princeton University, Princeton, NJ, United States; ^2^Department of Sociology and Criminology and Population Research Institute, The Pennsylvania State University, University Park, PA, United States

**Keywords:** mortality, life expectancy, smoking, inequality, urban-rural differences

## Abstract

**Background:**

Geographic inequality in US mortality has increased rapidly over the last 25 years, particularly between metropolitan and nonmetropolitan areas. These gaps are sizeable and rival life expectancy differences between the US and other high-income countries. This study determines the contribution of smoking, a key contributor to premature mortality in the US, to geographic inequality in mortality over the past quarter century.

**Methods:**

We used death certificate and census data covering the entire US population aged 50+ between Jan 1, 1990 and Dec 31, 2019. We categorized counties into 40 geographic areas cross-classified by region and metropolitan category. We estimated life expectancy at age 50 and the index of dissimilarity for mortality, a measure of inequality in mortality, with and without smoking for these areas in 1990–1992 and 2017–2019. We estimated the changes in life expectancy levels and percent change in inequality in mortality due to smoking between these periods.

**Results:**

We find that the gap in life expectany between metros and nonmetros increased by 2.17 years for men and 2.77 years for women. Changes in smoking-related deaths are responsible for 19% and 22% of those increases, respectively. Among the 40 geographic areas, increases in life expectancy driven by changes in smoking ranged from 0.91 to 2.34 years for men while, for women, smoking-related changes ranged from a 0.61-year decline to a 0.45-year improvement. The most favorable trends in years of life lost to smoking tended to be concentrated in large central metros in the South and Midwest, while the least favorable trends occurred in nonmetros in these same regions. Smoking contributed to increases in mortality inequality for men aged 70+, with the contribution ranging from 8 to 24%, and for women aged 50–84, ranging from 14 to 44%.

**Conclusions:**

Mortality attributable to smoking is declining fastest in large cities and coastal areas and more slowly in nonmetropolitan areas of the US. Increasing geographic inequalities in mortality are partly due to these geographic divergences in smoking patterns over the past several decades. Policies addressing smoking in non-metropolitan areas may reduce geographic inequality in mortality and contribute to future gains in life expectancy.

## Introduction

American mortality is undergoing an unprecedented stagnation. Since 2010, life expectancy gains have been among the slowest on record for the US, and life expectancy declined for three consecutive years between 2014 and 2017. Between 2010 and 2018, life expectancy increased by less than a tenth of a year (1–3). Considerable geographic variation underlies these national-level trends, with poor performance concentrated in nonmetropolitan areas and specific regions of the country. Some parts of the country—coastal areas and big cities—continue to post robust increases in life expectancy, while others—rural areas and the South and Appalachia—experience much slower rates of improvement (4, 5).

The divide between metropolitan and nonmetropolitan areas has grown considerably between 1990 and the present. While American cities of the early 1990s faced a number of social and economic dilemmas that limited their capacities for promoting healthy and long lives, the situation today is quite different. While they still face difficulties relating primarily to issues of equality, metropolitan areas today tend to have better outcomes along several dimensions, including educational attainment, public health infrastructure and outreach, and economic activity (4). Nonmetropolitan areas have experienced either slower improvement or deterioration along these same dimensions (6). In short, metropolitan areas have prospered while nonmetropolitan areas have been left behind, and this has been manifested in widening metro/nonmetro gaps in mortality.

Mortality inequalities are among the starkest manifestations of inequity in our society. Prior research suggests geographic inequality in mortality has increased over time, and that these inequalities have reached substantial magnitudes (4, 7–11). Where people live influences what policies are in place, their access to and quality of health care, the social and economic conditions they experience, and what health behaviors they practice. These differences are the most commonly proposed explanations for geographic inequalities and their growth over time (4, 7–12).

**Figure 1:**
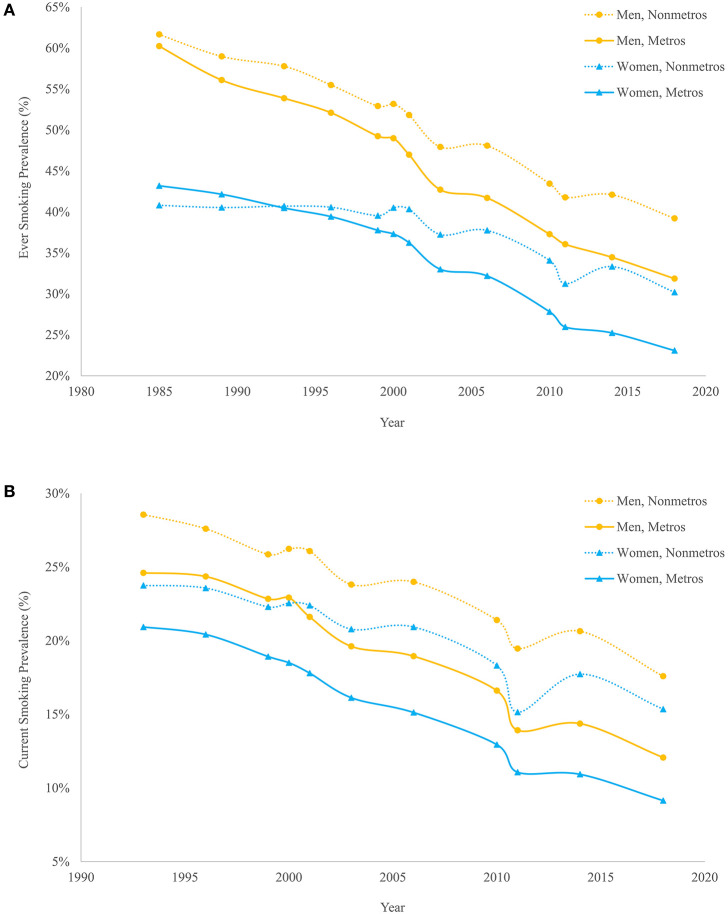
Ever smoking **(A)** and current smoking **(B)** prevalence by metropolitan status and sex, 1985–2018. Estimates are authors' calculations based on Current Population Survey Tobacco Use Supplements, 1985–2018. All estimates are based on the population aged 18 and older and are standardized to the 2000 U.S. population age distribution.

Cigarette smoking, the leading cause of premature morbidity and mortality in the United States, is one potential explanation that reflects all of the above dimensions. There is a vast literature that relates smoking to elevated levels of mortality and large and growing inequalities in mortality along a number of dimensions (13–20), and smoking is known to have contributed to past mortality variation among states. However, there has been relatively little research on the role of smoking-attributable mortality in explaining the metro-nonmetro divergence in U.S. mortality since the early 1990s. Figure 1 shows that while ever smoking and current smoking prevalence were similar between metros and nonmetros in the early 1990s, they have diverged significantly since then. Recent findings that cardiovascular disease, respiratory diseases, and lung cancer are among the key causes of death contributing to rising geographic inequality in mortality (10) also support the hypothesis that smoking likely plays a role in the growth in metro-nonmetro inequality in mortality over the last quarter century.

This study investigates the contribution of smoking to geographic inequality in mortality over the past three decades. Because of the rapidly growing gap between metropolitan and nonmetropolitan areas, we examine mortality inequalities across four metropolitan categories: large central metros, large metro suburbs, small and medium metros, and nonmetropolitan areas. We additionally explore whether the contribution of smoking to metro/nonmetro mortality inequalities is reproduced across ten regions of the country, since smoking-attributable mortality is known to have a strong regional component (21). These analyses shed light on how differences in smoking are contributing to divergent patterns of life expectancy gains across the nation, and in particular to the adverse mortality trends concentrated in nonmetropolitan areas.

## Materials and methods

### Data

We used the 1990–2019 National Center for Health Statistics (NCHS) Multiple Cause of Death data files, consisting of all deaths occurring in the US. The files contain information on decedents' age, sex, cause of death, and county of residence. These data were combined with Census population estimates to produce all-cause and lung cancer (ICD-9 code 162 for 1990–1998 and ICD-10 codes C33-C34 for 1999–2019) death rates by age, sex, geographic area (described below), and year. We considered four analytic periods: 1990–1992, 2000–2002, 2010–2012, and 2017–2019, with most analyses focusing on the first and last period. We focus on these specific periods because the metro-nonmetro mortality divergence commenced in the early 1990s (4, 7). Supplementary analyses (Supplementary Tables S1, S2; Supplementary Figure S1) show that our conclusions hold whether we use either 1990–1992 or 2000–2002 as the baseline period.

Both region and metropolitan/nonmetropolitan residence are key dimensions of geographic inequalities in mortality. To classify counties into metropolitan categories, we used codes developed by the US Department of Agriculture Economic Research Service, which were modified and made available by the NCHS. We used four categories: large central metros, large metro suburbs, small/medium metros, and nonmetropolitan areas. We considered 10 regions: New England, Middle Atlantic, East North Central, West North Central, South Atlantic, East South Central, West South Central, Mountain, Pacific, and Appalachia. The first nine regions were defined using the Census division categorization, while Appalachian counties were defined by the Appalachian Regional Commission classification. Appalachia consists of all of West Virginia and selected counties from 12 other states, which were excluded from their overlapping Census divisions. Supplementary Table S12 shows the correspondence between Census Region, Census Division, and state. For a subset of analyses, we cross-classified counties by region and metropolitan/nonmetropolitan category to identify 40 distinct geographic units. This 40-category classification has been used in prior studies and captures important features of geographic variation in mortality (4, 10).

### Analytic approach

Smoking is causally linked to many chronic diseases, including cardiovascular diseases, respiratory diseases, and cancers (22). In order to capture the total burden of smoking-related mortality, we used an indirect estimation method fitted to US data (21, 23). The method uses excess lung cancer mortality as an indicator of the damage caused by smoking, where excess lung cancer mortality is calculated as the difference between observed lung cancer mortality and the level of lung cancer mortality we would expect to observe among non-smokers. The method then models all-cause mortality as a function of excess lung cancer mortality to produce estimates of the proportion of deaths attributable to smoking by age and sex. The method was developed for ages 50+ because the mortality impacts of smoking manifest primarily at these ages (23). According to the 2018 US life table, 93.9% of Americans can expect to survive to age 50, so this analysis covers the great majority of deaths in the population (2).

The main assumption of this method is that lung cancer mortality accurately proxies for the cumulative burden of smoking, which is likely to be the case since the majority of lung cancer deaths in industrialized societies are attributable to smoking (24). Because lung cancer mortality reflects multiple forms of tobacco smoking, including cigarette and cigar smoking, this assumption means that the indirect estimates capture the broader impact of smoking across multiple product classes. The method's key advantages are that it captures the total burden of smoking-related mortality and relies on vital statistics data rather than self-reported smoking data, which is subject to reporting biases, may not accurately reflect individuals' lifetime smoking histories, and often results in underestimates of smoking-related mortality. We provide additional detail on the methodological approach in the Appendix.

Other studies have used direct approaches to examine smoking-attributable mortality, typically regressing mortality on smoking status to obtain relative risks. While the fine geographic detail used in this study does not allow for replication using direct methods applied to public-use data, prior research covering similar time periods focusing on other subpopulations has found qualitatively similar trends when applying either direct or indirect methodologies (25).

We estimated smoking-attributable mortality by age, sex, period, and geographic area. We computed life expectancy at age 50 with and without smoking using life table techniques and examined the contribution of smoking to life expectancy at age 50 in 1990–1992 and 2017–2019 and the change in its contribution for both the four metro categories and the 40 areas defined above.

We used two measures to quantify inequality and smoking's effect on inequality in mortality. For analyses focusing on the four metro categories, we computed a gradient measure equal to the difference in life expectancy at age 50 between large central metros and nonmetros, both with and without smoking-attributable mortality. We also assessed the contribution of smoking to the change over time in the gradient. In ancillary analyses (not shown here), we computed the difference between nonmetro areas and both large metro suburbs and small/medium metros and found qualitatively similar results.

As a summary measure of mortality inequality across the 40 cross-classified areas, we computed the index of dissimilarity (ID), one of the most commonly used measures of spatial unevenness. We calculated the ID for mortality with and without smoking-attributable deaths in each period to determine how much smoking contributes to geographic inequality in each period and to changes in geographic inequality over time.

The ID is calculated as:


$${}_{n}^{\;}ID_{x}=\frac{1}{2}\sum_{i=1}^{N}\left|\frac{{}_{n}D_{x}^{i}}{{}_{n}\;D_{x}}-\frac{{}_{n}\;P_{x}^{i}}{{}_{n}\;P_{x}}\right|$$

where $\frac{{}_{n}D_{x}^{i}}{{}_{n}D_{x}}$ is the proportion of national deaths at ages *x* to *x*+*n* occurring in place *i*, $\frac{{}_{n}P_{x}^{i}}{{}_{n}P_{x}}$ is the proportion of the national population aged *x* to *x*+*n* that lives in place *i*, and *N* is the total number of places (*N* = 40 geographic units). The ID has previously been used to study residential segregation, occupational and social mobility, and geographic inequality in mortality (10, 26, 27). Its value ranges between 0 and 1, with 0 indicating absolute equality and 1 absolute inequality. Among the ID's useful properties are that it is symmetric, invariant to population size, and easily interpreted as the proportion of national deaths that would need to be reallocated to a different area to achieve geographic equality in mortality. The ID was calculated for each 5-year age group between 50–54 and 80–84, and for an open-ended age group (85+).

## Results

Over the past three decades, gains in life expectancy have differed dramatically between metropolitan and nonmetropolitan areas within the US. Metro areas of all types, particularly large central metros, experienced much more rapid gains in life expectancy than nonmetros. Men experienced sizeable life expectancy increases between 1990–1992 and 2017–2019, gaining 4.64 years in large central metros, 3.92 years in large metro suburbs, 3.10 years in small metros, and 2.47 years in nonmetros (Table 1). Women had more modest gains in life expectancy, with values ranging from 0.65 years in nonmetros to 3.42 years in large central metros.

**Table 1:** Life expectancy at age 50 with and without smoking-attributable mortality by sex and metropolitan category, 1990–2019.

	**Men**								
	**1990–1992**			**2017–2019**			**Change**		
	**Obs**	**NS**	**YLL**	**Obs**	**NS**	**YLL**	**Obs**	**NS**	**YLL**
Large central metro	26.41	29.40	2.99	31.05	32.34	1.30	4.64	2.94	−1.70
Large metro suburb	27.31	30.19	2.88	31.23	32.57	1.34	3.92	2.38	−1.54
Small/Medium metro	26.85	29.91	3.06	29.95	31.51	1.56	3.10	1.60	−1.50
Nonmetro	26.34	29.49	3.15	28.80	30.66	1.86	2.47	1.17	−1.29
Gradient	0.08	−0.08		2.24	1.68		2.17	1.77	
	(0.00, 0.15)	(−0.18, 0.01)		(2.17, 2.32)	(1.59, 1.77)		(2.07, 2.27)	(1.64, 1.89)	
**Contribution of smoking to widening of gradient**							**19%**		
	**Women**								
	**1990–1992**			**2017–2019**			**Change**		
	**Obs**	**NS**	**YLL**	**Obs**	**NS**	**YLL**	**Obs**	**NS**	**YLL**
Large central metro	31.51	33.11	1.59	34.93	36.24	1.30	3.42	3.13	−0.29
Large metro suburb	31.99	33.58	1.59	34.53	35.99	1.46	2.53	2.40	−0.13
Small/Medium metro	31.98	33.42	1.44	33.58	35.02	1.44	1.60	1.60	0.00
Nonmetro	31.84	33.11	1.26	32.49	34.07	1.58	0.65	0.97	0.32
Gradient	−0.33	0.00		2.44	2.16		2.77	2.16	
	(−0.41, −0.25)	(−0.09, 0.09)		(2.36, 2.52)	(2.07, 2.25)		(2.66, 2.88)	(2.04, 2.29)	
**Contribution of smoking to widening of gradient**								**22%**	

Obs = observed life expectancy at age 50; NS = life expectancy at age 50 if smoking-related mortality is eliminated; YLL = years of life expectancy at age 50 lost due to smoking-related deaths; and Gradient = difference in life expectancy at age 50 between the large central metro and nonmetro categories. Values in brackets below Gradient values are 95% confidence intervals. Standard errors for Obs and NS values are given in Supplementary Table S3.

Changes in smoking-attributable mortality are part of the explanation for both the sex and the metro/nonmetro divergences (Table 1). Years of life lost due to smoking were sizeable for men in 1990–1992, ranging from 2.88 to 3.15 years. However, these values were quite similar across metro categories. By 2017–2019, years of life lost due to smoking had declined significantly, but the declines were more rapid in metropolitan areas. In 2017–2019, men lost between 1.30 years to smoking in large central metros and 1.86 years to smoking in nonmetros. Over this period, changes in smoking-attributable mortality were responsible for as much as 1.70 years of the 4.64-year life expectancy gain in large central metros and as little as 1.29 years of the 2.47-year life expectancy gain in nonmetros. All three types of metropolitan areas—large central metros, large metro suburbs, and small metros—experienced more rapid improvements from declines in smoking-related mortality than nonmetros.

The story for women is quite different. Unlike men, women initially experienced a reverse metro/nonmetro gradient in smoking-attributable mortality. In 1990–1992, women in nonmetros lost the fewest years of life to smoking (1.26 years). Women in large central metros lost the most years of life to smoking (1.59 years). By 2017–2019, this situation completely reversed, so that women in nonmetros lost the most years to smoking (1.58 years) while women in large central metros lost the fewest years to smoking (1.30 years). A very clear gradient emerged, wherein nonmetros experienced the greatest increase in years of life lost due to smoking, while the remaining three metropolitan areas experienced either no change or decreases in years of life lost to smoking.

The differential patterns in years of life lost to smoking by metro category contributed to an increase in inequality as measured by the difference in life expectancy between large central metros and nonmetros. The gradient increased by 2.17 years for men, and 19% of that increase was due to smoking. For women, the gradient between large central metros and nonmetros increased by 2.77 years, and 22% of that increase was due to smoking.

**Figure 2:**
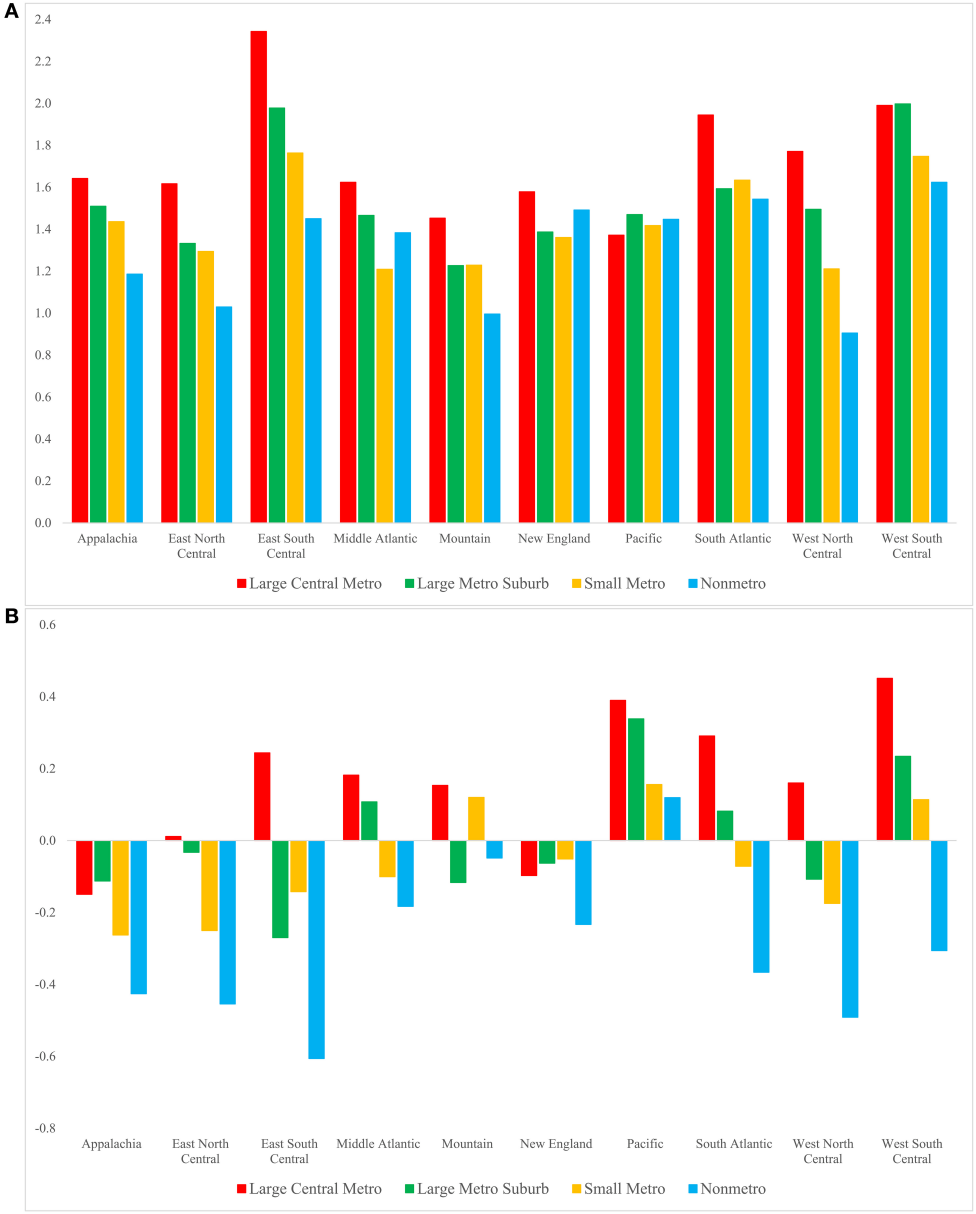
Contribution of the change in smoking-attributable mortality to the change in life expectancy at age 50 for men **(A)** and women **(B)** by region and metropolitan category, 1990–92 to 2017–19. Positive/negative values indicate that changes in smoking-attributable mortality contributed to an increase/decrease in life expectancy. These values are YLLs as defined in Table 1. Standard errors are given in Supplementary Tables S5, S6.

Considering finer geographic areas, we see that reductions in smoking-attributable mortality contributed to sizeable gains in life expectancy among men (Figure 2). This trend is evident across all regions and metropolitan/nonmetropolitan categories. The largest life expectancy improvements related to smoking occurred in large central metros in the Southern regions—East South Central (2.34 years), West South Central (1.99 years), and South Atlantic (1.94 years) —and in large metro suburbs in West South Central (2.00 years) and East South Central (1.98 years). The smallest improvements related to smoking were recorded in four nonmetropolitan areas: the Appalachian (1.19 years), East North Central (1.03 years), Mountain (1.00 years), and West North Central regions (0.91 years). Smoking-related improvements in male life expectancy at age 50 were most pronounced in large central metros and least pronounced in nonmetropolitan areas in the majority (six of the ten) of the regions. This pattern of differential life expectancy gains has contributed to a divergence between metropolitan and nonmetropolitan areas.

Life expectancy at age 50 increased for women across all geographic areas; however, most of these increases would have been considerably larger had smoking-attributable mortality not increased. We refer to this negative effect of smoking on longevity as smoking-related declines in life expectancy. Most regions experienced smoking-related declines in female life expectancy. The most notable exception to this pattern is the Pacific region, which experienced smoking-related improvements in life expectancy, with the largest improvement in large central metros (0.39 years) and the smallest improvement in nonmetros (0.12 years). Most nonmetros and small metros experienced smoking-related declines in female life expectancy between 1990–1992 and 2017–2019. These declines were most notable in nonmetropolitan areas of the East South Central (−0.61 years), West North Central (−0.49 years), East North Central (−0.45 years), and Appalachian (−0.43 years) regions. In contrast, nearly all large central metros and some large metro suburbs experienced life expectancy gains due to changes in smoking. These improvements were, however, much smaller than those among men, ranging from 0.01 to 0.45 years.

### Changes in the index of dissimilarity

**Figure 3:**
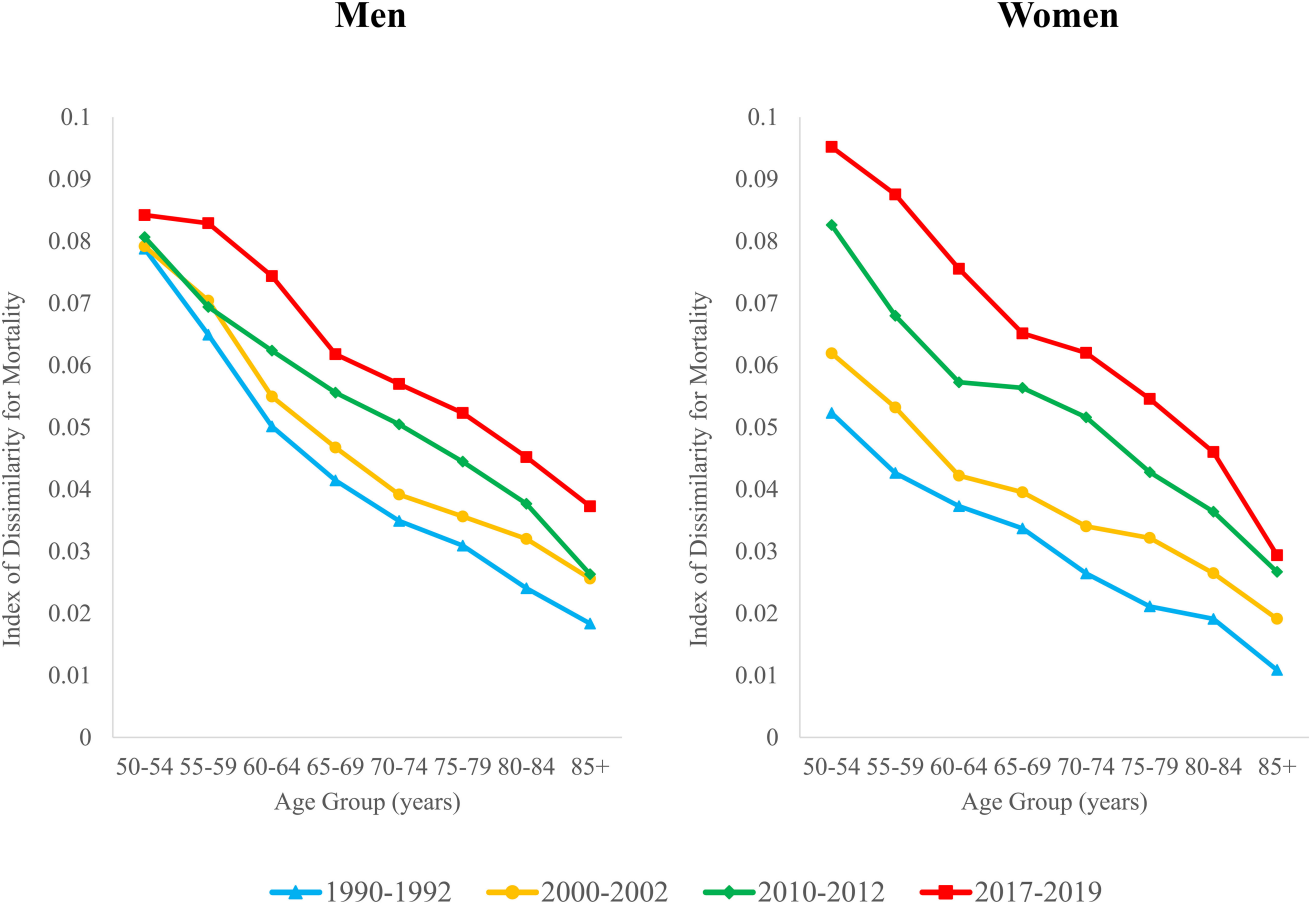
Geographic inequality in mortality by age and sex, 1990–1992 through 2017–2019.

Geographic inequality in mortality increased at all ages above 50 between 1990–1992 and 2017–2019 (Figure 3). Geographic inequality tends to be highest at younger ages but increased more rapidly over time at the older ages. Among men, the ID increased by between 7 and 49% at ages 50–69, while increases in the ID ranged from 63 to 103% for men aged 70 and older. For women, inequality nearly doubled at ages 50–69, and more than doubled at ages 70 and older. The increase in inequality was thus more pronounced for women across all ages. Whereas, geographic inequality was significantly lower for women than for men in the early 1990s, by 2017–2019, inequality was higher for women than for men at each age except 85+.

Table 2 shows values for the ID in 1990–1992 and 2017–2019, the contribution of smoking to the ID, and the contribution of smoking to the change over time in the ID. For men, the smoking contribution was highest in the early 1990s, ranging from 32 to 38% between ages 55–74 (column 5). While the percent contribution of smoking to the ID decreased over time, it still remained sizeable in 2017–2019, ranging from 20 to 24% for ages 55–74 (column 6). For women, there was a complete reversal in the percent contribution of smoking to the ID. In the 1990s, smoking contributed negatively to geographic inequality at all ages (smoking deaths reduced geographic inequality either because they were more evenly distributed than other causes of death or because they were negatively correlated with other causes of death across areas). By 2017–2019, smoking contributed to inequality in every age group except 85+, ranging between 11 and 18% at ages 50–79 (column 6). The smoking contribution tends to be larger at ages 50–79 and diminishes at the older ages. In both periods, smoking contributes to greater inequality for men than for women.

**Table 2:** Contribution of smoking-related deaths to geographic inequality in mortality by sex and age, 1990–2019.

		**Index of dissimilarity** **for mortality (ID)**		**Contribution of** **smoking to ID**		**% contribution of** **smoking to ID**		**% change in ID** **due to smoking^a^**
		**(1)**	**(2)**	**(3)**	**(4)**	**(5)**	**(6)**	**(7)**
	Age group	1990–1992	2017–2019	1990–1992	2017–2019	1990–1992	2017–2019	1990–2019
Men	50–54	0.079	0.084	0.019	0.012	24%	14%	−117% (−141, −94%)
	55–59	0.065	0.083	0.023	0.017	35%	20%	−33% (−37, −29%)
	60–64	0.050	0.074	0.018	0.015	36%	21%	−11% (−12, −9%)
	65–69	0.041	0.062	0.016	0.015	38%	24%	−4% (−5, −2%)
	70–74	0.035	0.057	0.011	0.013	32%	23%	8% (6, 9%)
	75–79	0.031	0.052	0.007	0.009	21%	17%	10% (9, 11%)
	80–84	0.024	0.045	0.003	0.005	10%	12%	13% (12, 14%)
	85+	0.018	0.037	0.000	0.004	−3%	11%	24% (23, 25%)
Women	50–54	0.052	0.095	−0.001	0.011	−2%	12%	28% (25, 31%)
	55–59	0.043	0.088	−0.004	0.016	−9%	18%	44% (42, 46%)
	60–64	0.037	0.076	−0.003	0.010	−7%	13%	33% (32, 34%)
	65–69	0.034	0.065	−0.001	0.007	−3%	11%	27% (25, 28%)
	70–74	0.026	0.062	−0.004	0.008	−14%	13%	32% (32, 33%)
	75–79	0.021	0.055	−0.003	0.006	−14%	11%	27% (26, 28%)
	80–84	0.019	0.046	−0.002	0.002	−9%	5%	14% (13, 15%)
	85+	0.011	0.029	−0.001	−0.001	−5%	−5%	−5% (−5, −4%)

^a^Percent change in ID between 1990–1992 and 2017–2019 due to smoking-attributable deaths, calculated as [(4) – (3)]/[(2) – (1)]. Values in parentheses are 95% confidence intervals.

Next, we assess the contribution of smoking deaths to changes over time in the ID between 1990–1992 and 2017–2019 (column 7). A negative value indicates that smoking tended to decrease inequality, while a positive value indicates that smoking deaths increased inequality. Changes in smoking-attributable mortality contributed to decreased inequality among men aged 50–69. At ages 70+, smoking contributed to increased geographic inequality for men. Its contribution was particularly large for the 85+ age group, where it accounted for 24% of the increase in inequality (column 7). In contrast, smoking contributed to increased geographic inequality for women in all age groups except 85+. Smoking was responsible for between 27 and 44% of the increase in inequality for women aged 50–79 (column 7).

## Discussion

Concerns about mortality stagnation and growing social and economic disparities have generated renewed interest in geographic inequality in American mortality. In this study, we find that smoking has become a major contributor to metropolitan-nonmetropolitan inequality, accounting for approximately one-fifth of the widening of the gap in life expectancy between large central metros and nonmetropolitan areas. This pattern holds for both men and women. This finding builds on prior research showing that geographic inequality in mortality increased in recent decades due to a range of causes of death and that smoking plays a role in regional differences in mortality (5, 10, 21, 28). We further show that the uneven geographic distribution of changes in smoking-attributable mortality is driving a substantial portion of the increase in inequality in life expectancy in the US. Large central metros and their suburbs are reaping the benefits of rapid reductions in smoking-attributable deaths, while nonmetros are being left behind.

These differential patterns have important implications for geographic inequality, which has increased substantially over the past three decades. Smoking-attributable deaths are responsible for roughly one-third of the increase in geographic inequality in mortality for women aged 50–79 when considering geographic units defined by regions cross-classified by metro category. For men, 10–24% of the increase among those aged 75+ was due to smoking. While smoking-attributable deaths constitute a larger proportion of overall deaths for men than for women, there was a greater geographic divergence in smoking patterns for women that drove their larger increase in inequality. This sex differential in the effects of smoking is largely a result of the differential patterns of smoking initiation and cessation for men vs. women. Men's smoking peaked in the 1950s, while women's smoking peaked approximately 15 years later (29). Thus, men's smoking-attributable mortality peaked in the 1990s and women's in the 2000s.

Much of the recent literature on mortality inequality has focused on contemporaneous phenomena as determinants of inequality. Smoking, on the other hand, is an exposure whose mortality effects accumulate over time and manifest decades later, often with a 20- to 40-year lag. The increases in geographic inequality in mortality today are due to changes in smoking behaviors that largely took place in the 1980s through the early 2000s. Even as smoking-attributable mortality declines, the uneven geographic patterning of those declines has contributed to growing geographic inequality.

While the popularity of cigarette smoking for the nation as a whole followed a pattern of rapid uptake followed by decline over the course of the 20th century, this process has occurred unevenly within the country across regions and between metro and nonmetro areas. The earliest data on smoking patterns date from the mid-1950s and suggest that smoking prevalence was higher in metropolitan than nonmetropolitan areas. At this time, the difference in smoking prevalence between urban and rural farm residents was around 11% points for men (52 vs. 41%) and 16% points among women (26 vs. 10%) (30, 31). Regional variation in smoking prevalence was fairly muted among men during this period, although heavy smoking was most common in the Northeast (31, 32). By the mid-1980s, however, smoking had become heavily concentrated in the South among men. The East South Central (35.8%), South Atlantic (34.4%), and West South Central (33.9%) regions had the highest percentage of men who were current smokers, while the Pacific region had the lowest percentage (27.7%) (33). Among women, regional differences were smaller. Smoking prevalence was highest in East North Central (27.5%), New England (25.9%), and the South Atlantic (25.3%), and lowest in the Pacific (22.4%) (33). There was also a reversal in the metro/nonmetro gradient as smoking prevalence declined in metro areas but either increased or stayed the same in nonmetro areas from the mid-1990s through the early 2000s (34). Between the mid-2000s and 2014, smoking declined in nonmetro areas but at much slower rates than in urban areas (30). For the past decade, current smokers in nonmetro areas and regions of the South have been more likely to have begun smoking at earlier ages (i.e., younger than age 16) and to smoke more cigarettes per day (35, 36). Today, nonmetros and parts of the South and Midwest are regarded as lagging far behind the rest of the nation in terms of their progress in reducing smoking and smoking-attributable mortality.

Several factors are thought to contribute to these patterns. These include: fewer and later adoption of tobacco control policies; the countering influence of the tobacco industry, particularly in tobacco-growing areas concentrated in the South; limited access to smoking cessation programs and interventions; and socioeconomic conditions of these areas. Tobacco control policies encompass a spectrum of policies such as excise taxes, media campaigns, and restrictions on smoking in public places and have been found to be effective in reducing smoking prevalence (37). However, nine of the ten states with the lowest excise taxes in 2011 were located in the Midwest (East and West North Central) and the South (South Atlantic, West South Central, and East South Central) (38, 39). Of the 24 states that lacked a comprehensive smoke-free law as of 2015, 17 were located in the South and Midwest (39). Studies have also suggested that tobacco control policies may be much more restricted in scope and less intense in nonmetropolitan areas (30, 40).

Weak tobacco regulations, particularly in tobacco-growing areas in the South, are thought to be related to their history of economic dependence on tobacco coupled with intensive tobacco industry influence. Nonmetros in these areas are viewed as having been particularly dependent on tobacco, and positive attitudes toward tobacco and smoking have persisted (30). Studies have found that in major tobacco growing regions, opposition to smoke-free laws and cigarette taxes was concentrated among tobacco farmers, hospitality associations, and tobacco companies. Tobacco companies sought to promote a pro-tobacco culture and block tobacco-control policies dating from the 1960s and continuing through the 1990s. These efforts included mobilizing farmers growing flue-cured tobacco in the South to block cigarette tax increases, highlighting the benefits that tobacco has brought to these economies, and emphasizing the threat tobacco-control policies pose to farmers and tax revenues (41). One example comes from the Philip Morris publication Smokers Advocate, which included the following as part of an “action alert” to oppose a cigarette tax hike in 1990: “At a time when tobacco is increasingly under attack throughout the rest of the country, North Carolinians need to “circle the wagons” and protect the economic future of as important a crop as tobacco” (42). The RJ Reynolds company created a “Pride in Tobacco” program in the late 1970s that focused on opposing tobacco-control policies in North Carolina, South Carolina, Wisconsin, Ohio, Kentucky, Virginia, and Tennessee. It continued operating through the 1990s (41). As a result, tobacco-growing parts of the South have been much slower to adopt tobacco-control policies, and when they do adopt them, they are more limited (e.g., less comprehensive coverage of workplaces, restaurants, and bars and lower taxes).

Both smoking initiation and cessation influence the risk of dying from a smoking-related cause of death and a population's level of smoking-attributable mortality. Nonmetro areas and regions of the South and Midwest have experienced poor socioeconomic conditions, in part related to deindustrialization. Studies have highlighted that lower education levels and knowledge of the health risks of smoking may be more prevalent in these areas (30). Low absolute and relative levels of education have been tied to high levels of mortality in the U.S. (43, 44). Poor socioeconomic conditions and daily life stressors may lead to smokers continuing to smoke as a form of stress relief (30). Qualitative studies of rural areas have documented a lack of support for quitting smoking within rural social networks (45). They have also found that the lack of alternative activities in nonmetro areas coupled with few public smoking bans and exposure to other smokers leads to both smoking initiation and continued smoking (45, 46). Economic constraints and limited access to smoking cessation programs and interventions also pose barriers to smoking cessation in these areas. Coverage for smoking cessation treatment services remains low, and some rural smokers have reported perceiving that buying cigarettes is less expensive than purchasing smoking cessation aides (34, 45). Smokers in nonmetropolitan areas may face particular challenges due to lack of smoking cessation programs in their local area, lack of mass media messaging about smoking prevention and treatment, and lack of knowledge of existing resources (34, 45). This is reflected in low use rates of smoking cessation aides such as nicotine lozenges, inhalers, or sprays or smoking cessation counseling in rural areas (45, 47).

Another class of explanations for the diverging life expectancy trends driven by smoking is selection. The populations of metropolitan and nonmetropolitan parts of the country have undergone significant change related to selective migration. More educated, healthier, well-to-do individuals have tended to leave nonmetropolitan areas in favor of large metros and their suburbs, meaning that those left behind in nonmetropolitan areas are likely to be negatively select on these same characteristics. Since education, underlying health, and income and wealth all tend to be negatively associated with both smoking and mortality, this form of selective migration is likely to lead to faster improvements in metropolitan life expectancy and either slower improvements or worsening of life expectancy in nonmetropolitan areas. Cigarette smoking uptake also tends to be concentrated in the teen years, so one's childhood place of residence may matter just as much as where one currently resides. Because of the likelihood that selective migration may be driving some of the trends documented in this study, the results cannot be interpreted as indicative of current place of residence driving 100% of the observed trends. Rather, a host of factors, including migration histories of a place's current population, determines mortality trends.

The main strengths of our study include the use of death certificate data covering the entire US population and the use of an indirect estimation method that captures the full burden of mortality associated with cigarette smoking. There are also several limitations to our study. It is possible that the relationship between lung cancer and all-other-cause mortality has changed over time, which would alter our results. This would be possible if, for example, mortality from causes unrelated to smoking has decreased over time, leading to a tighter, more positive relationship between lung cancer and all-other-cause mortality. We compute ancillary estimates taking into account this change and find that it only minimally influences our findings and does not change our substantive conclusions. Another potential concern is that our study focuses on ages 50+ and thus excludes smoking-related deaths below age 50. While prior research has shown that the smoking-attributable fraction is highly similar for ages 35+ relative to ages 50+ (23), we cannot rule out that smoking may also be important in explaining geographic variation in mortality below age 50. Estimates of the effect of smoking on life expectancy at birth that do not take into account smoking-attributable under-50 mortality are reported for the various geographic units in Supplementary Tables S7, S8. A third limitation is that we use only two measures of inequality: the metro/nonmetro life expectancy gap and the index of dissimilarity. It is possible that other measures of geographic inequality may yield different estimates. Supplementary analyses (Supplementary Table S9) indicate that our conclusions hold whether we use the index of dissimilarity or other measures of inequality, including the Gini coefficient and Theil's index. Finally, this article examines how increasing inequality is tied to smoking and does not examine how the contribution of smoking to geographic inequality might be related to racial and socioeconomic inequalities identified in other studies (25, 48–50). These social inequalities may act as mechanisms linking smoking and geographic inequality in mortality, as more vulnerable groups tend to have higher smoking-related mortality and are more concentrated in high-mortality regions. We find that in some regions of the country, rural areas lag behind in efforts to reduce smoking-attributable mortality. If, in those regions, racial and ethnic minorities are disproportionately concentrated in rural areas, we may expect within-region racial/ethnic disparities to persist or widen. The impacts of widening urban-rural inequality on racial/ethnic disparities and vice versa are nevertheless difficult to predict, since the composition of these areas has also changed over time, likely in a manner that is selective on latent traits predictive of mortality.

In debates surrounding inequality and mortality, researchers have often cast increasing inequality as a natural consequence of improvements in life expectancy. The most advantaged are able to reap the benefits of new knowledge and technologies, which in turn leads to increased inequality (51). What this study adds to the existing literature is identification of metropolitan status as a key dimension along which inequalities in smoking-attributable mortality have emerged over the past three decades. Differences between metropolitan and nonmetropolitan areas are complex and not easily captured by socioeconomic variables alone. Metropolitan status is a distinctly place-based categorization that encompasses differences between areas in their demographic, socioeconomic, environmental, cultural, and health system characteristics (52). For example, the legacy of economic dependence on tobacco and intensive tobacco industry influence has contributed to positive attitudes toward smoking and slower and more limited adoption of tobacco control policies in tobacco-growing nonmetro areas in the South (30, 40, 41). It is not simply that people in nonmetropolitan areas are poorer or less educated, but rather that the characteristics of these places themselves may lead to a greater burden of smoking-attributable mortality.

The results of this paper suggest that a number of policies can be implemented that would both increase life expectancy and reduce geographic inequality. Cigarette taxes tend to be higher in regions like the Northeast, which are also the areas where smoking-attributable mortality has declined the most. They tend to be lowest in states with large rural populations. In additional analyses (Supplementary Table S10), we find that states with lower cigarette tax rates experienced a greater metro-nonmetro divergence over time in years of life lost to smoking relative to states with higher taxes. Implementing higher cigarette taxes in areas like the South and the Midwest has the potential to reduce geographic inequality and metro/nonmetro inequality in mortality and to contribute to further gains in life expectancy (39, 53). Another potential set of policies encompasses comprehensive smoke-free laws for public areas. States that have not adopted these laws also tend to be concentrated in the South and hold a disproportionate share of the rural population (39). Similarly, tobacco retailer density and tobacco marketing has become more concentrated in rural parts of the country (54, 55). States with large rural populations can implement policies that would restrict retail tobacco growth, which would likely have the effect of decreasing nonmetropolitan smoking rates at the national level. Given the lag between smoking initiation or cessation and the mortality effects of smoking, the impacts of instituting any of these policies on reducing inequality would play out in the decades following the implementation of the policies.

While some policies like cigarette taxation tend to have the effect of reducing inequalities, others tend to do the opposite. This may be because of differential implementation, enforcement, and access to resources that make these programs less effective in nonmetropolitan areas. For example, one Kentucky-based study showed that smoke-free laws had different impacts on air quality due to differential enforcement (56). Nonmetros tend to have fewer smoking cessation programs and interventions, and tobacco control policies tend to be more restricted in scope in these areas (30, 40, 57). This would suggest that the federal and state governments should explore the possibility of targeting policies and smoking cessation resources specifically toward nonmetropolitan areas in order to reduce the disproportionate burden of smoking-attributable mortality in nonmetros.

Though the imprint of cigarette smoking on mortality is diminishing, new substances have emerged with the potential to drive new health inequalities. According to the 2020 National Youth Tobacco Survey, one-fifth of high school students are current users of e-cigarettes, up from roughly one-tenth in 2017 (58, 59). E-cigarette use is more common in rural areas at the national level, though there are important regional variations (60). The long-term health impacts of e-cigarette use are not yet well-established, and it is possible e-cigarettes could become new sources of premature mortality and inequalities in mortality in future decades. Other substances, like marijuana delivered through e-cigarettes, could also have long-term effects on mortality. On the other hand, these products may displace traditional cigarettes and thus have countervailing effects on smoking-attributable mortality (61, 62). Future trends in geographic inequality in mortality may be shaped by these new health behaviors, much as today's trends in inequality were partly shaped by the smoking behavior of cohorts in decades past. The findings of this paper and the emergence of these new technologies underscore the need for continued monitoring and coordinated efforts to prevent the uptake of potentially-deleterious health behaviors.

## Data availability statement

The original contributions presented in the study are included in the article/Supplementary materials, further inquiries can be directed to the corresponding author/s.

## Author contributions

AH and JH designed the study, analyzed and interpreted the data, and drafted and revised the manuscript. AH was responsible for submitting the manuscript for publication. All authors contributed to the article and approved the submitted version.

## Funding

This research was supported by grants from the Eunice Kennedy Shriver National Institute of Child Health and Human Development (R00 HD083519 and P2C HD047879) and the National Institute on Aging (R01 AG060115).

## Conflict of interest

The authors declare that the research was conducted in the absence of any commercial or financial relationships that could be construed as a potential conflict of interest.

## Publisher's note

All claims expressed in this article are solely those of the authors and do not necessarily represent those of their affiliated organizations, or those of the publisher, the editors and the reviewers. Any product that may be evaluated in this article, or claim that may be made by its manufacturer, is not guaranteed or endorsed by the publisher.
