# Supplementary material for: Smoking and the widening inequality in life expectancy between metropolitan and nonmetropolitan areas of the United States
Source: Front Public Health. 2022 Sep 7;10:942842. doi: 10.3389/fpubh.2022.942842 (PMC9490306; doi:10.3389/fpubh.2022.942842)
Supplement: Supplementary file 1 [file Data_Sheet_1.PDF]

Supplementary Material

1 Methodological Appendix

1.1 Indirect Estimation of Smoking-Attributable Mortality

We use an indirect estimation method developed by Preston, Glei, and Wilmoth(1, 2) and modified by Fenelon and Preston(3) to estimate smoking-attributable mortality. This method is similar to the one developed by Peto et al.(4) in that excess lung cancer mortality (i.e., lung cancer mortality in excess of what we would expect to observe among never-smokers) is used as an indicator of a cohort's cumulative exposure to smoking. Lung cancer mortality is a highly suitable proxy for the burden of smoking within a population given that it reflects prevalence, intensity, and duration of smoking and that over 90% and 70% of lung cancer deaths among men and women, respectively, are attributable to smoking.(5) It is well-established that cigarette smoking is the most important risk factor for lung cancer, and that variation in lung cancer incidence and mortality is primarily driven by variation in smoking behavior.(2, 6, 7)

The fraction of lung cancer deaths attributable to smoking in a given age-sex group, A_L, is calculated as:

$$A_L = \frac{M_L - \lambda_L^N}{M_L}$$

where M_L is the lung cancer death rate in that age-sex group and λ_L^N is the lung cancer death rate observed among non-smokers in that age-sex group in the Cancer Prevention Study II (CPS-II) for the period 1982-1988.(8) The lung cancer death rates among never-smokers (λ_L^N) used in this analysis are reported in Table A1 below.

Statistical modeling is used to estimate the fraction of all other deaths (i.e., all causes of death except lung cancer) attributable to smoking. Preston et al.(1, 2) estimated models using country-year-sex-age-group data from 20 high-income countries over the period 1950-2003. The authors estimated a negative binomial regression where the log of the death rate from all other causes was modeled as a function of age group (5-year categories), year (single-year dummies), country (categorical), the lung cancer death rate, and interactions between year (linear) and country, the lung cancer death rate and year (linear), and the lung cancer death rate and age group (categorical). The coefficients on the lung cancer death rate and interaction terms are combined to estimate the fraction of all other deaths attributable to smoking. Separate regressions were estimated for men and women.

Fenelon and Preston(3) followed the same approach but estimated the model using vital statistics data from U.S. states from 1996-2004. They estimated the following negative binomial regression model:

$$\ln(M_O) = \beta_L M_L + \beta_a X_a + \beta_s X_s + \beta_t t + \beta_{aL}(M_L \times X_a) + e$$

where M_O is the death rate from all other causes and M_L is the lung cancer death rate in a given state-year-sex-age group. X_a is age group, X_s is state, and t is year. For each age-sex group, the model coefficients on the lung cancer death rate are combined as follows:

$$\beta' = \beta_L + \beta_{aL}.$$

The β' coefficient estimates used in this analysis are reported in Table A1 below.

A_O, the fraction of all other deaths (i.e., non-lung cancer deaths) attributable to smoking in a given age-sex group, is calculated as:

$$A_O = 1 - e^{-\beta'(M_L - \lambda_L^N)},$$

The overall fraction of deaths from all causes attributable to smoking in a given age-sex group, A, is

$$A = \frac{A_L D_L + A_O D_O}{D}$$

where D_L, D_O, and D are deaths from lung cancer, all other causes except lung cancer, and all causes combined, respectively.

The counterfactual death rate (i.e., the death rate if smoking-attributable mortality were eliminated) in a given age-sex group is:

$$m^{-s} = m(1 - A)$$

where m is the observed death rate from all causes combined. These counterfactual death rates are used to calculate life expectancy without smoking. The contribution of smoking to life expectancy gradients is determined by comparing the life expectancy difference based on the observed death rates to the life expectancy difference based on the counterfactual death rates.

For each period in our analysis, we compute A and m^{-s} using observed age-sex-area-specific lung cancer deaths and death rates and all-cause deaths and death rates combined with the parameters given in Table A1 below. We then compute life expectancies based on both the observed (m) and counterfactual (m^{-s}) age-sex-area-specific death rates for each period. We additionally compute the index of dissimilarity across our 40 geographic units using both m and m^{-s} values to determine the contribution of smoking to geographic inequality in mortality for each age-sex group in each period.

We conducted a separate sensitivity analysis to examine the potential impact of any changes in the relationship between lung cancer mortality and all-other-cause mortality. In particular, the sensitivity analysis used the Preston et al. (1) approach in place of the Fenelon and Preston (3) approach, since the former includes a lung cancer-time interaction term in the negative binomial regression, which allows for the relationship between lung cancer and all-other-cause mortality to vary over time. The trend is in the form of an interaction term between year (measured as a linear variable) and lung cancer mortality rate, and the estimate for the coefficient is 0.0003 for men and 0.0010 for women (2). We apply this approach using the time trend and find that our estimates do not greatly differ. The qualitative findings and conclusions all remain the same.

Mortality rates (both cause-specific and all-cause) were computed at the county or super-county level using digitized death certificate records maintained by the National Center for Health Statistics' (NCHS) Mortality Statistics Branch and Vintage 2019 Bridged-Race Postcensal Population Estimates produced by the U.S. Census Bureau for the NCHS. Delta method standard errors or confidence intervals for all index of dissimilarity and life expectancy estimates are reported either in the main text or in an appendix table.

SUPPLEMEMTARY TABLE A1 | Lung cancer death rates among never-smokers (λ_L^N) and combined lung cancer regression coefficients (β') by sex and age group.

	Lung cancer death rates among Never-smokers (per 1,000)(8)		Model coefficients for lung cancer death rate (per 1,000)(1, 3)	
Age	Men	Women	Men	Women
50-54	0.06	0.06	0.297	0.207
55-59	0.05	0.07	0.186	0.175
60-64	0.12	0.12	0.111	0.087

65-69	0.22	0.17	0.073	0.085
70-74	0.35	0.31	0.046	0.069
75-79	0.52	0.33	0.027	0.056
80-84	0.89	0.58	0.016	0.039
85+	0.87	0.61	0.030	0.055

1.2 Current and Ever Smoking Prevalence Time Series Estimates

Data on ever and current smoking prevalence by metropolitan/nonmetropolitan status come from the Tobacco Use Supplement to the Current Population Survey (TUS-CPS) from the IPUMS database.(9) The TUS-CPS asked questions about ever smoking in 1985 and 1989 and in most individual years from 1992 forward, and questions about current smoking were asked in most years from 1992 forward. We produce population-weighted estimates using all available survey cycles. Estimates are grouped into individual years by computing the midpoint of each survey cycle. The estimates are based on respondents aged 18+ and age-standardized to the 2000 U.S. population SEER standard.(10)

1.3 Metropolitan and Region Category Coding

We assign each of 3,126 counties (or county equivalents) to a specific metropolitan category and a specific region. Our metropolitan categorization is defined using the 2013 NCHS Urban-Rural Classification Scheme, which was developed using the Office of Management and Budget's (OMB) categorization of counties into metropolitan statistical areas and micropolitan statistical areas. The OMB categorization in turn is based on a classification scheme originally developed by the Economic Research Service of the United States Department of Agriculture (USDA ERS). We use four metro categories: Large Central Metro, Large Metro Suburb, Small/Medium Metro, and Nonmetro. The correspondence between our four categories and the

USDA ERS classification is given in Appendix Table A2 below. Four counties were classified by the ERS as having fewer than 1 million people by were nevertheless classified as being in large metropolitan areas by the NCHS. This is due to the NCHS's use of updated population estimates from 2012. Because the OMB/NCHS categorization scheme does not include a category for suburbs of small/medium metros, we cannot separately examine trends in those suburbs. Prior research has shown that mortality levels and trends are highly similar in small and medium metros, contributing to our decision to combine small and medium metros into a single category (11). Large central metros and their suburbs, however, have very distinct mortality trends, so we separate out those two categories.

Counties are classified into regions on the basis of the U.S. Census Bureau's census division classification along with the Appalachian Regional Commission's classification of Appalachian counties. We move Appalachian counties from their census division classification into the Appalachia region so that each county is classified into exactly one region category among ten: New England, Middle Atlantic, East North Central, West North Central, South Atlantic, East South Central, West South Central, Mountain, Pacific, Appalachia. The reason we separate out Appalachian counties is that this region is historically, culturally, geographically, and socioeconomically distinct from the other counties that make up the census divisions. The types of industries and patterns of infrastructure development are far more similar within Appalachia than between Appalachian counties and their corresponding census divisions. For example, Fayette County, Pennsylvania is more similar to Taylor County, West Virginia than it is to Montgomery County, Pennsylvania, despite both Fayette and Montgomery Counties lying in the Middle Atlantic census division and Taylor County lying in the South Atlantic census division. Past work on health behaviors has shown distinctive patterns of health behavior uptake

in Appalachia compared to counties in neighboring census divisions. Separating out Appalachia is not an arbitrary decision—indeed, past research on health and health inequalities has highlighted the importance of Appalachia as a distinct region.(11-13)

SUPPLEMETARY TABLE A2 | Cross-tabulation of number of counties by USDA ERA rural-urban continuum code and metro category.

USDA ERS 2013 Rural-Urban Continuum Code	Metro Category Code: Large Central Metro	Large Metro Suburb	Small/ Medium Metro	Nonmetro	Total
Counties in metro areas of 1 million population or more	67	363	0	0	430
Counties in metro areas of 250,000 to 1 million population	1	3	372	0	376
Counties in metro areas of fewer than 250,000 population	0	0	355	0	355
Urban population of 20,000 or more, adjacent to a metro area	0	0	0	214	214
Urban population of 20,000 or more, not adjacent to a metro area	0	0	0	93	93
Urban population of 2,500 to 19,999, adjacent to a metro area	0	0	0	592	592
Urban population of 2,500 to 19,999, not adjacent to a metro area	0	0	0	431	431
Completely rural or less than 2,500 urban population, adjacent to a metro area	0	0	0	220	220
Completely rural or less than 2,500 urban population, not adjacent to a metro area	0	0	0	415	415
Total	68	366	727	1965	3,126

Note: Each cell displays the number of counties, and the total number of counties is 3,126. Metro categorization is based on the 2013 NCHS Urban-Rural Classification Scheme for Counties, which uses population estimates from July 1, 2012 to classify counties by metro area population. The USDA ERS 2013 Rural-Urban Continuum Codes use population estimates from the 2010 Census to classify counties by metro area population.

1.4 Methodological Appendix References

1. Preston SH, Glei DA, Wilmoth JR. Contribution of Smoking to International Differences in Life Expectancy. In: Crimmins EM, Preston SH, Cohen B, editors. *International Differences in Mortality at Older Ages: Dimensions and Sources*. Washington, DC: The National Academies Press (2011). p. 105-31.
2. Preston SH, Glei DA, Wilmoth JR. A New Method for Estimating Smoking-Attributable Mortality in High-Income Countries. *Int J Epidemiol* (2010) 39(2):430-8. Epub 2009/12/25. doi: 10.1093/ije/dyp360.
3. Fenelon A, Preston SH. Estimating Smoking-Attributable Mortality in the United States. *Demography* (2012) 49(3):797-818. doi: 10.1007/s13524-012-0108-x.
4. Peto R, Boreham J, Lopez AD, Thun M, Heath C. Mortality from Tobacco in Developed Countries: Indirect Estimation from National Vital Statistics. *The Lancet* (1992) 339(8804):1268-78. doi: https://doi.org/10.1016/0140-6736(92)91600-D.
5. Ezzati M, Lopez AD. Estimates of Global Mortality Attributable to Smoking in 2000. *The Lancet* (2003) 362(9387):847-52. doi: 10.1016/s0140-6736(03)14338-3.
6. Cheng TY, Cramb SM, Baade PD, Youlden DR, Nwogu C, Reid ME. The International Epidemiology of Lung Cancer: Latest Trends, Disparities, and Tumor Characteristics. *J Thorac Oncol* (2016) 11(10):1653-71. Epub 2016/07/02. doi: 10.1016/j.jtho.2016.05.021.
7. World Health Organization, International Agency for Research on Cancer. *Tobacco Smoke and Involuntary Smoking*. Lyon, France: IARC (2004).
8. Thun MJ, Day-Lally C, Myers DG, Calle EE, Flanders WD, Zhu B-P, et al. Trends in Tobacco Smoking and Mortality from Cigarette Use in Cancer Prevention Studies I (1959 through 1965) and Ii (1982 through 1988). In: Burns DM, Garfinkel L, Samet JM, editors. *Changes in Cigarette-Related Disease Risks and Their Implications for Prevention and Controll*. Tobacco Control Monograph No. 8. NIH Pub. No. 97-4213. Bethesda, MD: U.S. Department of Health and Human Services, National Institutes of Health, National Cancer Institute (1997). p. 305-82.
9. [Dataset] Flood S, King M, Rodgers R, Ruggles S, Warren JR, Westberry M. *Integrated Public Use Microdata Series, Current Population Survey: Version 9.0 [Dataset]*. (2021). Available: https://doi.org/10.18128/D030.V9.0.
10. SEER. Standard Populations (Millions) for Age-Adjustment (2021) [cited 2019 June 13]. Available from: https://seer.cancer.gov/stdpopulations/.
11. Elo IT, Hendi AS, Ho JY, Vierboom YC, Preston SH. Trends in Non-Hispanic White Mortality in the United States by Metropolitan-Nonmetropolitan Status and Region, 1990–2016. *Population and Development Review* (2019) 45(3):549-83. doi: https://doi.org/10.1111/padr.12249.
12. Singh GK, Kogan MD, Slifkin RT. Widening Disparities in Infant Mortality and Life Expectancy between Appalachia and the Rest of the United States, 1990–2013. *Health Affairs* (2017) 36(8):1423-32. doi: 10.1377/hlthaff.2016.1571.
13. Monnat SM. Trends in U.S. Working-Age Non-Hispanic White Mortality: Rural–Urban and within-Rural Differences. *Population Research and Policy Review* (2020) 39(5):805-34. doi: 10.1007/s11113-020-09607-6.

2 Supplementary Figures and Tables

2.1 Supplementary Figures

Supplementary Figure 1. Contribution of the change in smoking-attributable mortality to the change in life expectancy at age 50 by region and metropolitan category, 2000-2002 to 2017-19

Men

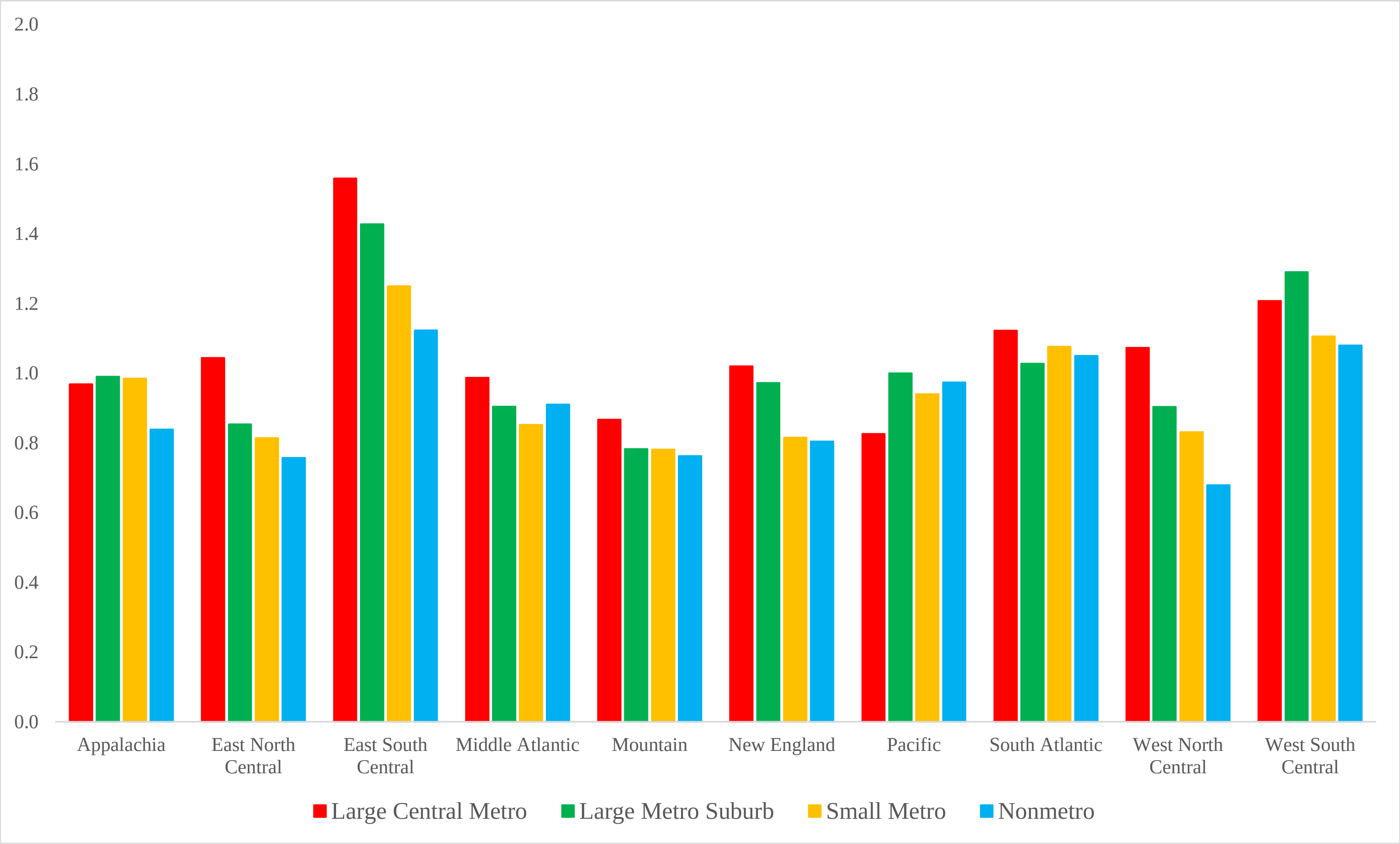

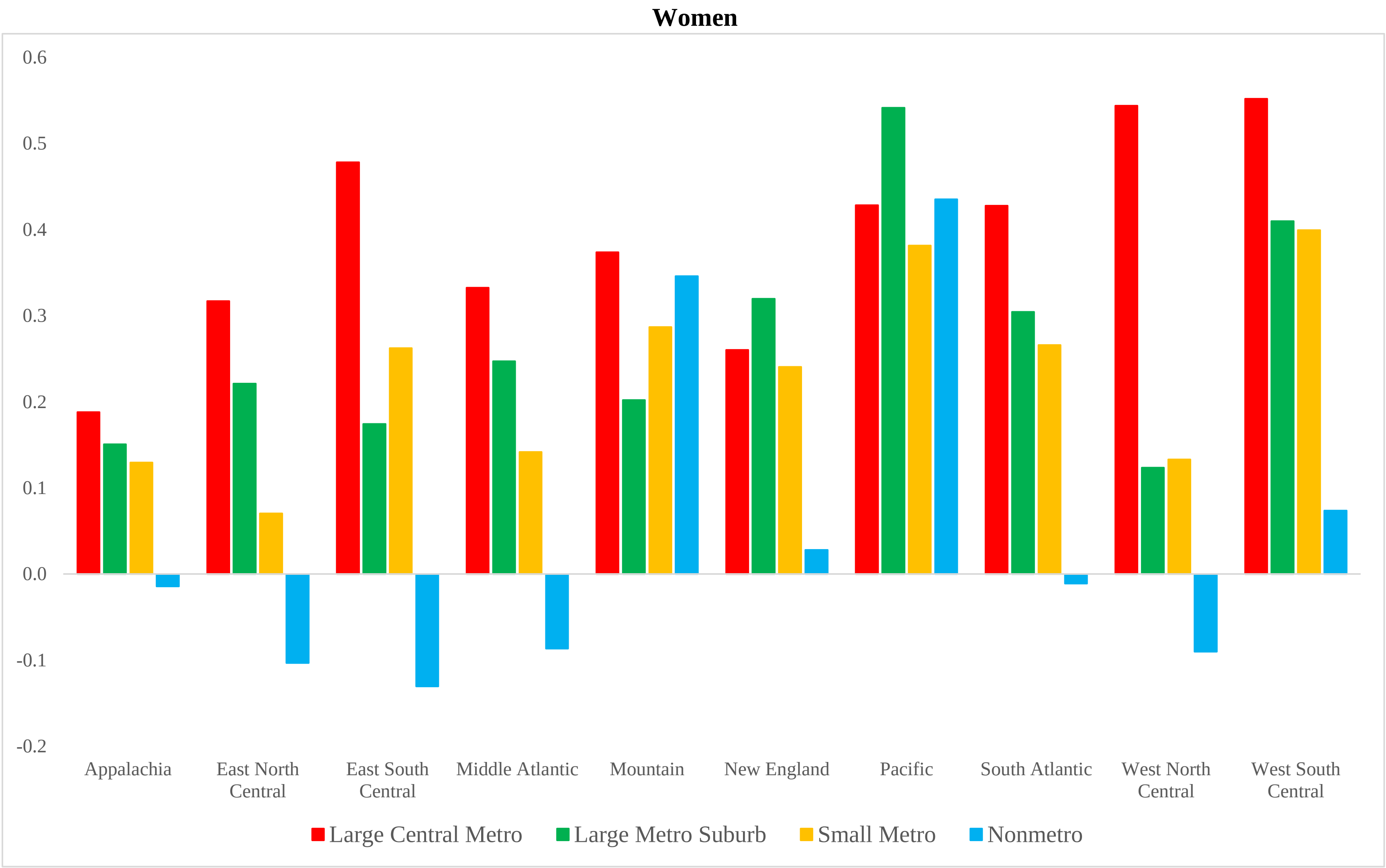

Note: Positive/negative values indicate that changes in smoking-attributable mortality contributed to an increase/decrease in life expectancy.

2.2 Supplementary Tables

Table S1. Life expectancy at age 50 with and without smoking-attributable mortality by sex and metropolitan category, 2000-2019

	Men									Women								
	2000-2002			2017-2019			Change			2000-2002			2017-2019			Change		
	Obs	NS	YLL	Obs	NS	YLL	Obs	NS	YLL	Obs	NS	YLL	Obs	NS	YLL	Obs	NS	YLL
Large Central Metro	28.20	30.54	2.34	31.05	32.34	1.30	2.85	1.81	-1.04	32.16	33.90	1.74	34.93	36.24	1.30	2.77	2.34	-0.43
Large Metro Suburb	28.80	31.15	2.35	31.23	32.57	1.34	2.43	1.42	-1.01	32.33	34.12	1.79	34.53	35.99	1.46	2.20	1.87	-0.33
Small Metro	28.21	30.76	2.55	29.95	31.51	1.56	1.74	0.75	-0.99	32.05	33.77	1.72	33.58	35.02	1.44	1.53	1.25	-0.28
Nonmetro	27.56	30.32	2.76	28.80	30.66	1.86	1.24	0.34	-0.90	31.68	33.29	1.61	32.49	34.07	1.58	0.82	0.79	-0.03
Gradient	0.64	0.22		2.24	1.68		1.61	1.46		0.49	0.61		2.44	2.16		1.95	1.55	
**Contribution of Smoking to Widening of Gradient**							**9%**									**21%**		

Note: Obs=observed life expectancy at age 50, NS=life expectancy at age 50 if smoking-related mortality is eliminated, YLL=years of life expectancy at age 50 lost due to smoking-related deaths, and Gradient=difference in life expectancy at age 50 between the large central metro and nonmetro categories.

Table S2. Contribution of smoking-related deaths to geographic inequality in mortality by sex and age, 2000-2019

		Index of Dissimilarity for Mortality (ID)		Contribution of Smoking to ID		% Contribution of Smoking to ID		% Change in ID Due to Smoking[a]
		(1)	(2)	(3)	(4)	(5)	(6)	(7)
	Age Group	2000-2002	2017-2019	2000-2002	2017-2019	2000-2002	2017-2019	2000-2019
Men	50-54	0.079	0.084	0.016	0.012	20%	14%	-70%
	55-59	0.070	0.083	0.018	0.017	25%	20%	-9%
	60-64	0.055	0.074	0.016	0.015	29%	21%	-3%
	65-69	0.047	0.062	0.015	0.015	32%	24%	-1%
	70-74	0.039	0.057	0.013	0.013	32%	23%	2%
	75-79	0.036	0.052	0.010	0.009	27%	17%	-5%
	80-84	0.032	0.045	0.005	0.005	17%	12%	-2%
	85+	0.026	0.037	0.004	0.004	14%	11%	4%
Women	50-54	0.062	0.095	0.001	0.011	2%	12%	30%
	55-59	0.053	0.088	0.002	0.016	4%	18%	41%
	60-64	0.042	0.076	-0.001	0.010	-3%	13%	35%
	65-69	0.040	0.065	0.000	0.007	0%	11%	28%
	70-74	0.034	0.062	0.002	0.008	7%	13%	19%
	75-79	0.032	0.055	0.001	0.006	2%	11%	24%
	80-84	0.026	0.046	-0.003	0.002	-12%	5%	28%
	85+	0.019	0.029	-0.004	-0.001	-19%	-5%	21%

[a] Percent change in ID between 2000-2002 and 2017-2019 due to smoking-attributable deaths, calculated as ((4) – (3)) / ((2) – (1))

Table S3. Standard Errors for Life Expectancy at Age 50 with and without Smoking-Attributable Mortality

With Smoking	Men		Women	
	1990-1992	2017-2019	1990-1992	2017-2019
Large Central Metro	0.024642	0.026891	0.025926	0.0269
Large Metro Suburb	0.028637	0.025163	0.030369	0.024987
Small Metro	0.023922	0.021639	0.026298	0.021635
Nonmetro	0.02623	0.026876	0.031102	0.02762

Without Smoking	Men		Women	
	1990-1992	2017-2019	1990-1992	2017-2019
Large Central Metro	0.032383	0.032071	0.029229	0.03218
Large Metro Suburb	0.038452	0.030385	0.034321	0.030705
Small Metro	0.031571	0.026122	0.028957	0.025909
Nonmetro	0.033593	0.0329	0.033482	0.03267

Table S4. Percent Contribution of Smoking to Change in Geographic Inequality in Mortality with 95% Confidence Intervals, 1990-1992 to 2017-2019

	Men			Women		
Age Group	Estimate	Lower CI	Upper CI	Estimate	Lower CI	Upper CI
50-54	-117%	-141%	-94%	28%	25%	31%
55-59	-33%	-37%	-29%	44%	42%	46%
60-64	-11%	-12%	-9%	33%	32%	34%
65-69	-4%	-5%	-2%	27%	25%	28%
70-74	8%	6%	9%	32%	32%	33%
75-79	10%	9%	11%	27%	26%	28%
80-84	13%	12%	14%	14%	13%	15%
85+	24%	23%	25%	-5%	-5%	-4%

Note: “Estimate” corresponds to the percent contribution of smoking to the change in the index of dissimilarity between 1990-1992 and 2017-2019. “Lower CI” and “Upper CI” are the lower and upper bounds of the 95% confidence interval, respectively.

Table S5. Smoking Contribution to Change in Life Expectancy at Age 50, Males

		Observed Life Expectancy				Life Expectancy Without Smoking				Effect of Smoking	
Metro	Region	1991	SE	2018	SE	1991	SE	2018	SE	Δ	SE
1	1	26.78	0.13	30.87	0.14	29.62	0.17	32.13	0.17	1.58	0.31
1	2	25.77	0.06	31.65	0.08	28.58	0.07	32.84	0.10	1.62	0.16
1	3	25.62	0.06	29.42	0.06	28.85	0.07	31.04	0.08	1.62	0.14
1	4	26.30	0.12	30.27	0.13	29.42	0.16	31.62	0.16	1.77	0.29
1	5	25.91	0.07	30.66	0.08	29.25	0.09	32.06	0.09	1.94	0.17
1	6	25.02	0.14	28.14	0.14	29.11	0.19	29.88	0.17	2.34	0.32
1	7	26.02	0.08	30.29	0.08	29.23	0.12	31.50	0.09	1.99	0.19
1	8	27.36	0.12	30.99	0.10	29.88	0.15	32.06	0.11	1.45	0.24
1	9	27.55	0.05	32.43	0.05	29.94	0.07	33.45	0.06	1.37	0.12
1	10	25.86	0.12	28.73	0.12	29.24	0.17	30.46	0.16	1.64	0.29
2	1	27.42	0.10	31.47	0.09	30.04	0.13	32.70	0.10	1.39	0.21
2	2	27.34	0.06	31.82	0.06	29.97	0.08	32.97	0.07	1.47	0.13
2	3	27.06	0.07	30.68	0.06	29.84	0.09	32.13	0.07	1.33	0.14
2	4	27.43	0.11	31.01	0.10	30.28	0.16	32.37	0.12	1.49	0.25
2	5	27.36	0.06	31.32	0.06	30.32	0.09	32.68	0.07	1.59	0.14
2	6	25.78	0.19	29.17	0.15	29.58	0.26	30.99	0.20	1.98	0.41
2	7	26.41	0.13	30.42	0.10	29.76	0.17	31.77	0.12	2.00	0.26
2	8	28.46	0.21	32.55	0.16	30.50	0.26	33.37	0.18	1.23	0.41
2	9	27.66	0.10	31.68	0.09	30.12	0.13	32.67	0.10	1.47	0.21
2	10	26.12	0.12	29.70	0.10	29.31	0.16	31.38	0.13	1.51	0.26
3	1	27.29	0.09	31.10	0.08	29.99	0.12	32.44	0.10	1.36	0.20
3	2	26.96	0.08	30.56	0.08	29.65	0.10	32.04	0.09	1.21	0.18
3	3	26.76	0.06	29.46	0.05	29.70	0.08	31.11	0.06	1.29	0.13
3	4	27.29	0.08	30.11	0.08	30.02	0.10	31.62	0.09	1.21	0.18
3	5	26.66	0.07	30.29	0.06	30.00	0.09	31.99	0.07	1.63	0.14
3	6	25.30	0.12	28.12	0.11	29.15	0.17	30.21	0.14	1.76	0.27
3	7	26.14	0.07	28.67	0.07	29.53	0.10	30.31	0.08	1.75	0.16
3	8	27.88	0.11	30.72	0.08	30.01	0.13	31.63	0.09	1.23	0.21
3	9	27.72	0.07	30.87	0.06	30.26	0.10	31.99	0.07	1.42	0.15
3	10	25.85	0.06	28.58	0.06	29.12	0.08	30.42	0.07	1.44	0.13
4	1	26.98	0.14	30.58	0.13	29.89	0.19	31.99	0.16	1.49	0.31
4	2	26.33	0.19	30.36	0.21	29.30	0.25	31.94	0.26	1.38	0.46
4	3	26.78	0.06	29.39	0.06	29.58	0.08	31.16	0.07	1.03	0.14
4	4	27.38	0.06	29.66	0.07	29.92	0.08	31.30	0.08	0.91	0.14
4	5	24.79	0.09	27.76	0.09	28.51	0.12	29.93	0.11	1.54	0.21
4	6	24.92	0.10	26.47	0.10	28.79	0.13	28.90	0.13	1.45	0.23
4	7	25.89	0.07	27.65	0.08	29.47	0.09	29.61	0.09	1.62	0.17
4	8	27.52	0.11	30.55	0.11	29.49	0.14	31.52	0.13	1.00	0.25
4	9	27.57	0.13	30.39	0.13	30.29	0.18	31.67	0.15	1.45	0.30
4	10	25.60	0.06	27.89	0.06	28.95	0.08	30.07	0.07	1.19	0.14

Note: The Δ under "Effect of Smoking" refers to the effect of smoking on the change in life expectancy between 1990-1992 and 2017-2019. "1991" and "2018" refer to the 1990-1992 and 2017-2019 periods, respectively. SE indicates standard errors that correspond to the preceding column. Metro categories are: 1=Large Central Metro, 2=Large Metro Suburb, 3=Small/Medium Metro, and 4=Nonmetro. Region categories are: 1= New England, 2=Middle Atlantic, 3=East North Central, 4=West North Central, 5=South Atlantic, 6=East South Central, 7=West South Central, 8=Mountain, 9=Pacific, 10=Appalachia.

Table S6. Smoking Contribution to Change in Life Expectancy at Age 50, Females

		Observed Life Expectancy				Life Expectancy Without Smoking				Effect of Smoking	
Metro	Region	1991	SE	2018	SE	1991	SE	2018	SE	Δ	SE
1	1	31.89	0.14	35.03	0.14	33.31	0.15	36.54	0.18	-0.10	0.31
1	2	30.91	0.06	35.83	0.08	32.32	0.07	37.05	0.09	0.18	0.15
1	3	30.71	0.06	33.47	0.06	32.31	0.07	35.05	0.08	0.01	0.13
1	4	31.65	0.13	34.23	0.14	33.28	0.14	35.69	0.17	0.16	0.29
1	5	31.42	0.08	34.86	0.08	33.01	0.09	36.16	0.09	0.29	0.16
1	6	30.38	0.15	32.48	0.14	32.26	0.17	34.12	0.17	0.24	0.31
1	7	31.05	0.09	33.99	0.08	32.63	0.10	35.11	0.09	0.45	0.18
1	8	32.14	0.14	34.59	0.10	33.55	0.15	35.85	0.12	0.15	0.25
1	9	31.97	0.05	36.25	0.06	33.50	0.06	37.39	0.07	0.39	0.12
1	10	30.80	0.13	33.05	0.13	32.32	0.15	34.72	0.16	-0.15	0.28
2	1	32.18	0.10	35.09	0.09	33.68	0.11	36.65	0.11	-0.06	0.20
2	2	31.69	0.06	35.25	0.06	33.23	0.07	36.68	0.07	0.11	0.13
2	3	31.44	0.07	33.82	0.06	32.92	0.08	35.33	0.07	-0.03	0.14
2	4	32.06	0.12	34.14	0.10	33.55	0.14	35.74	0.12	-0.11	0.24
2	5	31.99	0.07	34.84	0.06	33.55	0.08	36.31	0.07	0.08	0.14
2	6	30.92	0.21	32.52	0.15	32.42	0.23	34.29	0.19	-0.27	0.39
2	7	31.11	0.14	33.62	0.10	32.68	0.15	34.96	0.12	0.23	0.25
2	8	32.28	0.22	35.35	0.15	33.32	0.24	36.51	0.19	-0.12	0.41
2	9	31.82	0.11	35.07	0.09	33.47	0.12	36.39	0.11	0.34	0.21
2	10	31.17	0.13	33.06	0.10	32.48	0.15	34.48	0.12	-0.11	0.25
3	1	32.13	0.09	34.69	0.08	33.63	0.10	36.24	0.10	-0.05	0.19
3	2	31.74	0.09	34.15	0.08	33.15	0.10	35.65	0.09	-0.10	0.18
3	3	31.61	0.06	33.09	0.05	32.97	0.07	34.70	0.07	-0.25	0.13
3	4	32.49	0.09	33.77	0.08	33.76	0.10	35.21	0.09	-0.17	0.18
3	5	31.80	0.07	34.10	0.06	33.25	0.08	35.62	0.07	-0.07	0.14
3	6	30.84	0.13	31.99	0.11	32.30	0.15	33.60	0.13	-0.14	0.26
3	7	31.28	0.08	32.61	0.07	32.69	0.09	33.90	0.08	0.11	0.16
3	8	32.40	0.12	34.20	0.08	33.56	0.13	35.25	0.09	0.12	0.21
3	9	32.05	0.08	34.58	0.07	33.57	0.09	35.95	0.08	0.16	0.16
3	10	31.23	0.07	32.34	0.05	32.49	0.07	33.87	0.06	-0.26	0.13
4	1	31.93	0.16	34.11	0.14	33.28	0.17	35.70	0.17	-0.23	0.31
4	2	31.23	0.22	33.54	0.21	32.92	0.24	35.41	0.26	-0.18	0.47
4	3	31.91	0.07	32.88	0.06	33.11	0.08	34.54	0.07	-0.45	0.14
4	4	32.81	0.07	33.41	0.07	33.78	0.08	34.87	0.08	-0.49	0.15
4	5	30.60	0.10	31.92	0.09	31.79	0.11	33.48	0.10	-0.37	0.20
4	6	30.80	0.12	30.88	0.11	31.98	0.13	32.66	0.13	-0.61	0.24
4	7	31.41	0.08	31.53	0.08	32.74	0.09	33.17	0.09	-0.31	0.17
4	8	32.04	0.14	34.00	0.12	33.05	0.15	35.06	0.14	-0.05	0.28
4	9	31.77	0.15	34.04	0.13	33.51	0.18	35.67	0.17	0.12	0.32
4	10	31.19	0.07	31.59	0.06	32.44	0.08	33.27	0.07	-0.43	0.14

Note: The Δ under "Effect of Smoking" refers to the effect of smoking on the change in life expectancy between 1990-1992 and 2017-2019. "1991" and "2018" refer to the 1990-1992 and 2017-2019 periods, respectively. SE indicates standard errors that correspond to the preceding column. Metro categories are: 1=Large Central Metro, 2=Large Metro Suburb, 3=Small/Medium Metro, and 4=Nonmetro. Region categories are: 1= New England, 2=Middle Atlantic, 3=East North Central, 4=West North Central, 5=South Atlantic, 6=East South Central, 7=West South Central, 8=Mountain, 9=Pacific, 10=Appalachia.

Table S7. Smoking Contribution to Change in Life Expectancy at Birth, Males

		Observed Life Expectancy				Life Expectancy Without Smoking				Effect of Smoking	
Metro	Region	1991	SE	2018	SE	1991	SE	2018	SE	Δ	SE
1	1	72.25	0.16	77.59	0.18	74.79	0.21	78.76	0.22	1.37	0.39
1	2	68.64	0.07	78.42	0.11	70.98	0.09	79.53	0.13	1.23	0.20
1	3	70.13	0.07	74.95	0.08	72.96	0.09	76.41	0.10	1.37	0.17
1	4	71.46	0.15	76.34	0.17	74.24	0.20	77.58	0.21	1.55	0.37
1	5	69.82	0.08	76.98	0.10	72.69	0.11	78.28	0.12	1.58	0.21
1	6	69.92	0.16	72.88	0.18	73.53	0.24	74.41	0.22	2.07	0.40
1	7	70.75	0.10	76.90	0.10	73.57	0.14	78.03	0.12	1.69	0.23
1	8	72.66	0.14	77.33	0.12	74.91	0.19	78.31	0.15	1.27	0.30
1	9	72.80	0.06	79.80	0.07	74.94	0.08	80.76	0.08	1.17	0.15
1	10	71.20	0.15	73.70	0.16	74.22	0.21	75.24	0.20	1.47	0.37
2	1	74.38	0.11	78.43	0.11	76.82	0.16	79.58	0.14	1.29	0.26
2	2	73.82	0.07	78.57	0.07	76.24	0.10	79.65	0.09	1.34	0.16
2	3	73.65	0.08	77.18	0.07	76.23	0.11	78.52	0.09	1.23	0.18
2	4	74.09	0.14	77.44	0.13	76.74	0.20	78.70	0.15	1.39	0.31
2	5	73.21	0.08	77.54	0.07	75.90	0.11	78.80	0.09	1.43	0.18
2	6	71.79	0.23	75.04	0.19	75.25	0.32	76.69	0.25	1.80	0.51
2	7	72.12	0.15	77.09	0.13	75.16	0.22	78.35	0.15	1.78	0.33
2	8	74.91	0.25	79.38	0.21	76.80	0.32	80.14	0.25	1.13	0.52
2	9	73.67	0.12	78.78	0.11	75.92	0.16	79.71	0.13	1.32	0.26
2	10	72.24	0.14	75.87	0.13	75.16	0.19	77.41	0.17	1.37	0.32
3	1	73.70	0.11	77.50	0.11	76.19	0.15	78.73	0.13	1.25	0.25
3	2	73.42	0.10	76.86	0.10	75.90	0.13	78.23	0.12	1.11	0.22
3	3	73.03	0.07	75.48	0.07	75.74	0.10	77.00	0.08	1.20	0.16
3	4	73.81	0.10	76.83	0.10	76.33	0.13	78.24	0.12	1.11	0.23
3	5	71.83	0.08	75.89	0.07	74.81	0.11	77.43	0.09	1.43	0.18
3	6	70.58	0.14	73.65	0.14	74.01	0.20	75.52	0.18	1.56	0.34
3	7	71.58	0.09	74.67	0.08	74.62	0.12	76.17	0.10	1.55	0.20
3	8	73.79	0.13	76.81	0.10	75.73	0.16	77.65	0.12	1.12	0.26
3	9	73.69	0.09	77.54	0.08	76.01	0.12	78.58	0.10	1.27	0.19
3	10	71.71	0.07	74.12	0.07	74.68	0.10	75.77	0.09	1.31	0.17
4	1	73.68	0.17	76.65	0.18	76.37	0.23	77.94	0.21	1.40	0.40
4	2	73.01	0.23	76.93	0.28	75.75	0.30	78.40	0.34	1.27	0.58
4	3	73.04	0.08	75.49	0.08	75.61	0.10	77.11	0.10	0.95	0.18
4	4	73.51	0.08	75.79	0.09	75.85	0.10	77.29	0.11	0.83	0.19
4	5	69.14	0.11	72.97	0.12	72.37	0.15	74.91	0.15	1.29	0.27
4	6	69.37	0.12	71.46	0.13	72.75	0.17	73.59	0.16	1.24	0.29
4	7	70.99	0.09	73.15	0.10	74.18	0.12	74.91	0.12	1.43	0.21
4	8	72.77	0.14	75.99	0.15	74.55	0.17	76.87	0.17	0.89	0.32
4	9	72.88	0.17	76.31	0.17	75.33	0.23	77.47	0.20	1.29	0.38
4	10	71.12	0.07	73.23	0.08	74.14	0.10	75.17	0.10	1.08	0.17

Note: The Δ under "Effect of Smoking" refers to the effect of smoking on the change in life expectancy between 1990-1992 and 2017-2019. "1991" and "2018" refer to the 1990-1992 and 2017-2019 periods, respectively. SE indicates standard errors that correspond to the preceding column. Metro categories are: 1=Large Central Metro, 2=Large Metro Suburb, 3=Small/Medium Metro, and 4=Nonmetro. Region categories are: 1= New England, 2=Middle Atlantic, 3=East North Central, 4=West North Central, 5=South Atlantic, 6=East South Central, 7=West South Central, 8=Mountain, 9=Pacific, 10=Appalachia.

Table S8. Smoking Contribution to Change in Life Expectancy at Birth, Females

		Observed Life Expectancy				Life Expectancy Without Smoking				Effect of Smoking	
Metro	Region	1991	SE	2018	SE	1991	SE	2018	SE	Δ	SE
1	1	79.52	0.18	83.20	0.20	80.87	0.20	84.66	0.24	-0.11	0.41
1	2	77.27	0.08	84.01	0.11	78.57	0.09	85.19	0.13	0.12	0.20
1	3	77.60	0.08	80.87	0.08	79.10	0.09	82.37	0.10	-0.01	0.18
1	4	79.06	0.17	81.84	0.19	80.61	0.19	83.24	0.23	0.15	0.39
1	5	78.16	0.10	82.71	0.10	79.65	0.12	83.95	0.12	0.24	0.22
1	6	77.25	0.19	79.37	0.18	79.01	0.23	80.90	0.23	0.23	0.41
1	7	78.41	0.11	81.93	0.10	79.90	0.13	83.00	0.12	0.41	0.23
1	8	79.73	0.18	82.43	0.13	81.08	0.20	83.64	0.16	0.14	0.34
1	9	79.65	0.07	84.73	0.08	81.11	0.08	85.84	0.09	0.35	0.16
1	10	78.10	0.17	80.19	0.17	79.54	0.20	81.76	0.22	-0.14	0.38
2	1	80.35	0.13	83.43	0.12	81.80	0.15	84.94	0.15	-0.07	0.27
2	2	79.53	0.08	83.44	0.08	81.00	0.09	84.82	0.10	0.09	0.17
2	3	79.34	0.09	81.68	0.08	80.76	0.11	83.12	0.10	-0.03	0.19
2	4	80.12	0.16	82.04	0.13	81.56	0.19	83.58	0.17	-0.10	0.32
2	5	79.54	0.09	82.63	0.08	81.02	0.11	84.04	0.10	0.07	0.19
2	6	78.52	0.26	79.98	0.19	79.95	0.30	81.66	0.25	-0.25	0.51
2	7	78.82	0.17	81.63	0.13	80.32	0.20	82.92	0.16	0.21	0.33
2	8	80.28	0.29	83.39	0.21	81.28	0.32	84.51	0.26	-0.11	0.55
2	9	79.65	0.14	83.36	0.12	81.23	0.16	84.63	0.15	0.31	0.28
2	10	78.83	0.17	80.60	0.13	80.08	0.19	81.96	0.16	-0.10	0.33
3	1	80.08	0.12	82.62	0.12	81.51	0.14	84.10	0.14	-0.05	0.26
3	2	79.58	0.11	81.96	0.11	80.93	0.13	83.40	0.13	-0.10	0.24
3	3	79.30	0.08	80.55	0.07	80.60	0.09	82.08	0.09	-0.23	0.17
3	4	80.38	0.12	81.66	0.11	81.60	0.13	83.05	0.13	-0.17	0.25
3	5	78.91	0.09	81.33	0.08	80.27	0.11	82.77	0.09	-0.07	0.19
3	6	77.96	0.17	79.15	0.14	79.34	0.20	80.66	0.18	-0.13	0.34
3	7	78.63	0.10	80.04	0.09	79.96	0.12	81.27	0.11	0.11	0.21
3	8	80.00	0.15	81.85	0.11	81.11	0.17	82.85	0.13	0.11	0.29
3	9	79.72	0.10	82.56	0.09	81.17	0.12	83.87	0.11	0.14	0.21
3	10	78.76	0.09	79.45	0.07	79.97	0.10	80.89	0.09	-0.23	0.17
4	1	80.01	0.20	81.77	0.19	81.31	0.22	83.28	0.23	-0.21	0.42
4	2	79.00	0.28	81.22	0.29	80.62	0.32	83.01	0.36	-0.17	0.63
4	3	79.63	0.09	80.31	0.09	80.78	0.10	81.88	0.10	-0.42	0.19
4	4	80.41	0.10	80.90	0.10	81.34	0.11	82.29	0.11	-0.46	0.21
4	5	77.22	0.13	78.58	0.12	78.34	0.15	80.04	0.14	-0.34	0.27
4	6	77.42	0.16	77.42	0.14	78.52	0.17	79.07	0.17	-0.55	0.32
4	7	78.53	0.11	78.46	0.10	79.78	0.12	79.99	0.12	-0.28	0.23
4	8	79.41	0.18	81.10	0.17	80.37	0.20	82.10	0.19	-0.04	0.37
4	9	79.15	0.20	81.28	0.18	80.80	0.24	82.81	0.23	0.12	0.43
4	10	78.55	0.09	78.43	0.08	79.74	0.10	80.00	0.10	-0.38	0.19

Note: The Δ under "Effect of Smoking" refers to the effect of smoking on the change in life expectancy between 1990-1992 and 2017-2019. "1991" and "2018" refer to the 1990-1992 and 2017-2019 periods, respectively. SE indicates standard errors that correspond to the preceding column. Metro categories are: 1=Large Central Metro, 2=Large Metro Suburb, 3=Small/Medium Metro, and 4=Nonmetro. Region categories are: 1= New England, 2=Middle Atlantic, 3=East North Central, 4=West North Central, 5=South Atlantic, 6=East South Central, 7=West South Central, 8=Mountain, 9=Pacific, 10=Appalachia.

Table S9. Percent Contribution of Smoking to Change in Geographic Inequality in Mortality Using Alternate Inequality Metrics, 1990-1992 to 2017-2019

	Male			Female		
Age Group	Gini	Theil	ID	Gini	Theil	ID
50-54	-27%	10%	-117%	24%	29%	28%
55-59	4%	30%	-33%	36%	41%	44%
60-64	7%	31%	-11%	24%	29%	33%
65-69	1%	30%	-4%	28%	31%	27%
70-74	12%	35%	8%	33%	36%	32%
75-79	11%	28%	10%	24%	28%	27%
80-84	13%	22%	13%	13%	13%	14%
85+	26%	28%	24%	1%	1%	-5%
Correlation with ID	0.95	0.76	1.00	0.96	0.97	1.00

Note: The three inequality measures are the Gini index (Gini), Theil index (Theil), and the Index of Dissimilarity (ID), each of which is computed across the 40 area categories defined by four metro categories cross-classified by 10 regions. The final row gives the correlation between each index and the ID. All three measures are computed taking into account differential population sizes across geographic units.

Table S10. Gap between Large Central Metros and Nonmetros in Years of Life Lost to Smoking Above Age 50 by State Cigarette Tax Quintile, 1990-2019

	**Men**				
	Metro-Nonmetro e_{50} Gap Due to Smoking by Cigarette Tax Quintile				
Tax Quintile	1990-1992	2000-2002	2010-2012	2017-2019	Change 1990-2019
1	0.06	0.18	0.27	0.35	0.29
2	-0.15	0.12	0.31	0.30	0.44
3	0.00	0.32	0.52	0.49	0.49
4	-0.45	-0.09	-0.02	0.16	0.61
5	-0.05	0.45	0.77	0.79	0.84
	**Women**				
	Metro-Nonmetro e_{50} Gap Due to Smoking by Cigarette Tax Quintile				
Tax Quintile	1990-1992	2000-2002	2010-2012	2017-2019	Change 1990-2019
1	0.11	0.13	0.28	0.47	0.36
2	-0.35	-0.12	0.09	0.19	0.54
3	-0.36	-0.17	0.14	0.26	0.62
4	-0.38	-0.18	0.02	0.15	0.53
5	-0.56	-0.31	0.02	0.25	0.82

Note: Each tax quintile contains 10 states (50 total), with 1 and 5 indicating states with the highest and lowest cigarette taxes, respectively. Cigarette taxes are per pack of 20 cigarettes and are based on data from the July 2014 update of the State Health Policy Research Dataset (SHEPRD) for Public Use (Silver and Macinko 2014). Tax quintiles are computed using averaged tax rates for 1994-2010.

Table S11. Contribution of Smoking to Life Expectancy at Age 50 by Sex, Metropolitan Category, and Region, 1990-1992 to 2017-2019

	1990-1992			2017-2019			Smoking-Related Change in Life Expectancy
Men	With smoking	Without smoking	Years lost to smoking	With smoking	Without smoking	Years lost to smoking	
**Large Central Metro**	**26.41**	**29.40**	**2.99**	**31.05**	**32.34**	**1.30**	**1.70**
Appalachia	25.86	29.24	3.38	28.73	30.46	1.73	1.64
East North Central	25.62	28.85	3.23	29.42	31.04	1.62	1.62
East South Central	25.02	29.11	4.08	28.14	29.88	1.74	2.34
Middle Atlantic	25.77	28.58	2.81	31.65	32.84	1.19	1.62
Mountain	27.36	29.88	2.52	30.99	32.06	1.07	1.45
New England	26.78	29.62	2.84	30.87	32.13	1.26	1.58
Pacific	27.55	29.94	2.39	32.43	33.45	1.02	1.37
South Atlantic	25.91	29.25	3.34	30.66	32.06	1.40	1.94
West North Central	26.30	29.42	3.12	30.27	31.62	1.35	1.77
West South Central	26.02	29.23	3.21	30.29	31.50	1.22	1.99
**Large Metro Suburb**	**27.31**	**30.19**	**2.88**	**31.23**	**32.57**	**1.34**	**1.54**
Appalachia	26.12	29.31	3.19	29.70	31.38	1.68	1.51
East North Central	27.06	29.84	2.78	30.68	32.13	1.45	1.33
East South Central	25.78	29.58	3.79	29.17	30.99	1.82	1.98
Middle Atlantic	27.34	29.97	2.62	31.82	32.97	1.16	1.47
Mountain	28.46	30.50	2.04	32.55	33.37	0.81	1.23
New England	27.42	30.04	2.62	31.47	32.70	1.23	1.39
Pacific	27.66	30.12	2.46	31.68	32.67	0.99	1.47
South Atlantic	27.36	30.32	2.96	31.32	32.68	1.36	1.59
West North Central	27.43	30.28	2.86	31.01	32.37	1.36	1.49
West South Central	26.41	29.76	3.35	30.42	31.77	1.35	2.00
**Small/Medium Metro**	**26.85**	**29.91**	**3.06**	**29.95**	**31.51**	**1.56**	**1.50**
Appalachia	25.85	29.12	3.27	28.58	30.42	1.83	1.44
East North Central	26.76	29.70	2.95	29.46	31.11	1.65	1.29
East South Central	25.30	29.15	3.85	28.12	30.21	2.08	1.76
Middle Atlantic	26.96	29.65	2.69	30.56	32.04	1.48	1.21
Mountain	27.88	30.01	2.14	30.72	31.63	0.91	1.23
New England	27.29	29.99	2.70	31.10	32.44	1.34	1.36
Pacific	27.72	30.26	2.54	30.87	31.99	1.12	1.42
South Atlantic	26.66	30.00	3.33	30.29	31.99	1.70	1.63
West North Central	27.29	30.02	2.72	30.11	31.62	1.51	1.21
West South Central	26.14	29.53	3.39	28.67	30.31	1.64	1.75
**Nonmetropolitan**	**26.34**	**29.49**	**3.15**	**28.80**	**30.66**	**1.86**	**1.29**
Appalachia	25.60	28.95	3.36	27.89	30.07	2.17	1.19
East North Central	26.78	29.58	2.80	29.39	31.16	1.77	1.03
East South Central	24.92	28.79	3.87	26.47	28.90	2.42	1.45
Middle Atlantic	26.33	29.30	2.97	30.36	31.94	1.58	1.38
Mountain	27.52	29.49	1.97	30.55	31.52	0.97	1.00
New England	26.98	29.89	2.90	30.58	31.99	1.41	1.49
Pacific	27.57	30.29	2.72	30.39	31.67	1.28	1.45
South Atlantic	24.79	28.51	3.72	27.76	29.93	2.17	1.54
West North Central	27.38	29.92	2.54	29.66	31.30	1.64	0.91

West South Central	25.89	29.47	3.58	27.65	29.61	1.96	1.62

Women	1990-1992 With smoking	1990-1992 Without smoking	1990-1992 Years lost to smoking	2017-2019 With smoking	2017-2019 Without smoking	2017-2019 Years lost to smoking	Smoking-Related Change in Life Expectancy
**Large Central Metro**	**31.51**	**33.11**	**1.59**	**34.93**	**36.24**	**1.30**	**0.29**
Appalachia	30.80	32.32	1.52	33.05	34.72	1.67	-0.15
East North Central	30.71	32.31	1.60	33.47	35.05	1.59	0.01
East South Central	30.38	32.26	1.88	32.48	34.12	1.64	0.24
Middle Atlantic	30.91	32.32	1.40	35.83	37.05	1.22	0.18
Mountain	32.14	33.55	1.42	34.59	35.85	1.26	0.15
New England	31.89	33.31	1.41	35.03	36.54	1.51	-0.10
Pacific	31.97	33.50	1.53	36.25	37.39	1.14	0.39
South Atlantic	31.42	33.01	1.59	34.86	36.16	1.30	0.29
West North Central	31.65	33.28	1.63	34.23	35.69	1.47	0.16
West South Central	31.05	32.63	1.57	33.99	35.11	1.12	0.45
**Large Metro Suburb**	**31.99**	**33.58**	**1.59**	**34.53**	**35.99**	**1.46**	**0.13**
Appalachia	31.17	32.48	1.31	33.06	34.48	1.42	-0.11
East North Central	31.44	32.92	1.47	33.82	35.33	1.51	-0.03
East South Central	30.92	32.42	1.50	32.52	34.29	1.77	-0.27
Middle Atlantic	31.69	33.23	1.54	35.25	36.68	1.43	0.11
Mountain	32.28	33.32	1.04	35.35	36.51	1.16	-0.12
New England	32.18	33.68	1.50	35.09	36.65	1.57	-0.06
Pacific	31.82	33.47	1.65	35.07	36.39	1.31	0.34
South Atlantic	31.99	33.55	1.56	34.84	36.31	1.47	0.08
West North Central	32.06	33.55	1.50	34.14	35.74	1.60	-0.11
West South Central	31.11	32.68	1.57	33.62	34.96	1.34	0.23
**Small/Medium Metro**	**31.98**	**33.42**	**1.44**	**33.58**	**35.02**	**1.44**	**0.00**
Appalachia	31.23	32.49	1.26	32.34	33.87	1.53	-0.26
East North Central	31.61	32.97	1.36	33.09	34.70	1.61	-0.25
East South Central	30.84	32.30	1.46	31.99	33.60	1.61	-0.14
Middle Atlantic	31.74	33.15	1.41	34.15	35.65	1.51	-0.10
Mountain	32.40	33.56	1.17	34.20	35.25	1.05	0.12
New England	32.13	33.63	1.49	34.69	36.24	1.55	-0.05
Pacific	32.05	33.57	1.52	34.58	35.95	1.37	0.16
South Atlantic	31.80	33.25	1.44	34.10	35.62	1.52	-0.07
West North Central	32.49	33.76	1.27	33.77	35.21	1.45	-0.17
West South Central	31.28	32.69	1.41	32.61	33.90	1.29	0.11
**Nonmetropolitan**	**31.84**	**33.11**	**1.26**	**32.49**	**34.07**	**1.58**	**-0.32**
Appalachia	31.19	32.44	1.26	31.59	33.27	1.68	-0.43
East North Central	31.91	33.11	1.20	32.88	34.54	1.66	-0.45
East South Central	30.80	31.98	1.18	30.88	32.66	1.78	-0.61
Middle Atlantic	31.23	32.92	1.69	33.54	35.41	1.87	-0.18
Mountain	32.04	33.05	1.01	34.00	35.06	1.06	-0.05
New England	31.93	33.28	1.35	34.11	35.70	1.59	-0.23
Pacific	31.77	33.51	1.74	34.04	35.67	1.62	0.12
South Atlantic	30.60	31.79	1.20	31.92	33.48	1.56	-0.37
West North Central	32.81	33.78	0.97	33.41	34.87	1.47	-0.49
West South Central	31.41	32.74	1.33	31.53	33.17	1.64	-0.31

Table S12. Correspondence between Census Region, Census Division, and State

Census Region	Census Division	Corresponding States
Northeast	New England	CT, ME, MA, NH, RI, VT
	Middle Atlantic	NJ, NY, PA
Midwest	East North Central	IN, IL, MI, OH, WI
	West North Central	IA, KS, MN, MO, NE, ND, SD
South	South Atlantic	DE, DC, FL, GA, MD, NC, SC, VA
	East South Central	AL, KY, MS, TN
	West South Central	AR, LA, OK, TX
West	Mountain	AZ, CO, ID, NM, MT, UT, NV, WY
	Pacific	AK, CA, HI, OR, WA
-	Appalachia*	WV (all counties) and selected counties in AL, GA, KY, MD, MS, NY, NC, OH, PA, SC, TN, and VA that are excluded from the other 9 areas

* Appalachia is not a Census Division but is instead a separate region defined by the Appalachian Regional Commission. The nine Census Divisions and Appalachia together comprise the 10 "regions" used in this analysis.

2.3 Table References

Silver, Diana, and Macinko, James. State Health Policy Research Dataset (SHEPRD): 1980-2010. Inter-university Consortium for Political and Social Research [distributor], 2014-09-24. https://doi.org/10.3886/ICPSR34789.v3
